# Electrochemical Chlorine Shuttle from PVC Waste to Vinyl Ether Acceptors for the Synthesis of Biodegradable Polyester Precursors

**DOI:** 10.1002/adma.202517489

**Published:** 2025-11-12

**Authors:** Sebastian Becker, Dominik Wördehoff, Dominik Weis, Simon Horsinka, Siegfried R. Waldvogel, Pol Besenius

**Affiliations:** ^1^ Department of Chemistry Johannes Gutenberg University Mainz Duesbergweg 10–14 55128 Mainz Germany; ^2^ Department of Electrosynthesis Max‐Planck‐Institute for Chemical Energy Conversion Stiftstraße 34–36 45470 Mülheim an der Ruhr Germany; ^3^ Institute of Biological and Chemical Systems−Functional Molecular Systems (IBCS−FMS) Karlsruhe Institute of Technology Kaiserstraße 12 76131 Karlsruhe Germany

**Keywords:** chlorine transfer, dechlorination reaction, DoE, electrolysis, PVC, recycling

## Abstract

Poly(vinyl chloride) (PVC) remains one of the most widely produced polymers globally but poses significant environmental and energetic challenges due to its high chlorine content, low recyclability, and the energy‐intensive nature of both production and disposal. Here, a sustainable electrochemical approach is reported for the dechlorination of PVC and simultaneous synthetic generation of cyclic acetals, precursors for biodegradable polyesters, employing a Cl_2_‐shuttle reaction onto vinyl ethers. By systematic electrochemical screening and a Design of Experiments (DoE) optimization strategy, high yields of cyclic acetal formation and elevated dechlorination rates of up to 94% for PVC are achieved. The resulting polymer retains its structural integrity and exhibits internal plasticization via covalent recombination with phthalate plasticizer fragments. This shows that beyond chlorine recovery, the process opens up attractive upcycling pathways through tunable thermal properties of partially dechlorinated PVC. Additionally, our method is tolerant to common plastic additives and applicable to real PVC waste streams, highlighting its robustness and industrial potential. The broad scope of accessible cyclic acetals expands the utility of the reported process using PVC as a chlorine source, providing a platform to produce valuable monomer precursors and enabling facilitated access to versatile, biodegradable polyester materials.

## Graphical abstract

A sustainable, paired electrolysis method is presented to recycle PVC, one of the world's most used polymer materials. Our innovative and robust process transfers chlorine from waste PVC and produces valuable precursors for biodegradable polyester materials. The macromolecular architecture of the treated, dechlorinated PVC is preserved and exhibits internal plasticized thermal properties, opening up exciting upcycling possibilities.

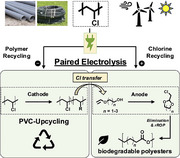

## 1 Introduction

The recycling of poly(vinyl chloride), the third most produced polymer material worldwide after polyethylene and polypropylene, remains a major challenge.^[^ ^1^ ^]^ Due to the extensive use of additives, reaching weight percentages of up to 60% in the final material formulation, and the high intrinsic chlorine content and its action as flame retardant, make energy recovery through combustion very difficult.^[^ ^2^, ^3^ ^]^ PVC recycling rates are extremely low, such as 3% in the US.^[^ ^4^ ^]^ End‐of‐life PVC is widely regarded as hazardous and an economically unattractive waste material that accumulates in landfills and still poses risks, such as leaching toxic additives.^[^ ^5^, ^6^, ^7^ ^]^ Non‐recycled PVC waste streams contribute to the material's extremely poor energy profile. The overall manufacturing process of PVC is endothermic, since the industrial production of chlorine via chlor‐alkali electrolysis is highly energy intensive and accounts for ≈2% of the total electricity consumption in Germany.^[^ ^8^, ^9^ ^]^ Nevertheless, on an industrial scale, chlorine chemistry is ubiquitous. It is essential in all sectors for the synthesis of solvents, catalysts, and key chemical building blocks. Notably, 90% of pharmaceuticals and 86% of crop protection chemicals contain chlorine, or chlorine chemistry is involved in their intermediates.^[^ ^10^ ^]^ The high energy content of chlorine enables the synthesis of acid chlorides (e.g., PCl_3_, SiCl_4_, COCl_2_), which provide an enormous thermodynamic driving force for downstream synthetic routes.^[^ ^11^ ^]^ Importantly, 39% of industrial chlorine production is used for PVC manufacturing, representing a major Cl_2_‐One‐Way‐Road towards chloro‐organic waste products.^[^ ^12^, ^13^ ^]^ Hence, there is a high demand for innovative chemical recycling strategies for PVC to establish a circular economy of chlorine on industrial scales.

The central concept in chemical PVC recycling involves the cleavage of the carbon‐chlorine (C─Cl) bond to recover both carbon scaffold and chlorine content separately. The high bond dissociation energy of the C─Cl bond combined with the macromolecular nature of the substrate, exhibits a significant challenge for the desired quantitative dechlorination of the polymer material.^[^ ^14^, ^15^ ^]^ Various strategies have been developed for the dechlorination of PVC using thermal/basic eliminations, catalytic pathways, or electrochemical reduction, many include sequential techniques to avoid polymer degradation or crosslinking.^[^ ^14^, ^16^, ^17^, ^18^, ^19^, ^20^, ^21^, ^22^, ^23^, ^24^, ^25^, ^26^, ^27^, ^28^, ^29^, ^30^, ^31^ ^]^ However, most of these approaches put large emphasis on polymer re‐ and/or upcycling only, and strategies to efficiently recover both the polymer scaffold and the chlorine content remain rare. Focusing on the dechlorination of PVC, catalytic approaches stand out compared to thermal eliminations, with high dechlorination rates enabling precise reaction control over the C─Cl bond cleavage and leading to quantitative dechlorination.^[^ ^17^, ^19^, ^20^, ^21^ ^]^ The applicability for industrial scales remains limited, however, since the used catalysts typically require inert conditions, are sensitive to plasticizers and stabilizers, operate at elevated temperatures or pose a severe cost factor.

Here, we propose that the electrochemical dechlorination of PVC via a paired electrolysis strategy represents a promising route to recover both chlorine and the polymer backbone efficiently, while retaining industrial scalability.^[^ ^32^ ^]^ Electrochemistry, particularly electro‐organic synthesis, combines the use of renewable energy and selective chemical reactivity.^[^ ^33^, ^34^, ^35^, ^36^ ^]^ Key benefits include the replacement of chemical reagents by electrical current, and the separation of halides or halogens, which are often gaseous and corrosive, is avoided. An ideal scenario would be the in situ transfer of halides from a stable feedstock like waste PVC, circumventing the use of chlorine.^[^ ^37^ ^]^ Numerous examples of halogen transfer in electro‐organic synthesis have been reported so far.^[^ ^32^, ^38^, ^39^, ^40^, ^41^, ^42^, ^43^, ^44^, ^45^, ^46^, ^47^, ^48^ ^]^ The electrochemical dihalogenation has a technical impact, wherein for sophisticated building blocks the dibromination is often preferred over the dichlorination.^[^ ^49^, ^50^ ^]^ The waldvogel and morandi labs reported conditions to use dibromo‐ or dichloroethane, the latter in combination with manganese catalysis, as halide sources which were transferred electrochemically to olefins, thus yielding new vicinal dichlorides or dibromides. Importantly, this chemistry is amenable to the use of γ–hexachlorocyclohexane (Lindane) as a potent chlorine donor. For example, 1‐dodecene could be chlorinated, obtaining high yields (91%) on a multigram scale (16 g) using Lindane as chlorine source while liberating benzene. Even soil samples artificially contaminated with Lindane could serve as a substrate, highlighting the robustness of this electrochemical upcycling strategy.^[^ ^48^ ^]^ A few examples on electrochemical re‐ and upcycling of PVC exist.^[^ ^22^, ^31^, ^51^, ^52^, ^53^ ^]^ Remarkably, mcneil and coworkers successfully harnessed the liberated chlorine from PVC in a direct oxidative chlorination of arenes. Additionally, they investigated the mediative properties of phthalate esters, plasticizers used in PVC, for electrochemical cleavage of the C─Cl bond.^[^ ^31^ ^]^ Despite the innovative paired electrolysis approach, the dechlorination rate of PVC remains low, and the practical relevance of chlorinated arenes reported at an industrial scale remains uncertain.

Given the vast amount of PVC waste, the danger of shifting the waste issue from PVC to the chlorine acceptor molecule is a significant concern. Consequently, finding innovative and industrially relevant chlorine acceptors is crucial for establishing truly circular material streams. One promising approach involves the electrochemical chlorination of vinyl ethers with a terminal hydroxy group, such as 4‐hydroxybutyl vinyl ether, yielding 2‐chloromethyl‐1,3‐dioxepane. This compound can be used for the manufacturing of poly(caprolactone) (PCL), a biodegradable polyester, via one step elimination and polymerization.^[^ ^54^, ^55^, ^56^ ^]^ Additionally, this monomer can also enhance the degradation profile of otherwise persistent commodity polymer materials. Due to the radical polymerization mechanism, ketene acetal monomers can be incorporated via drop‐in copolymerization into materials such as polystyrene (PS) and polymethyl methacrylate (PMMA).^[^ ^54^, ^57^, ^58^, ^59^ ^]^ This copolymerization introduces hydrolysable ester bonds in the polymer backbone that facilitate polymer degradation: this highlights the usefulness of the synthesized chlorine acceptor for multiple materials sectors on an industrial scale.^[^ ^56^ ^]^

## 2 Results and Discussion

### 2.1 Electrochemical Screening

**Figure 1:**
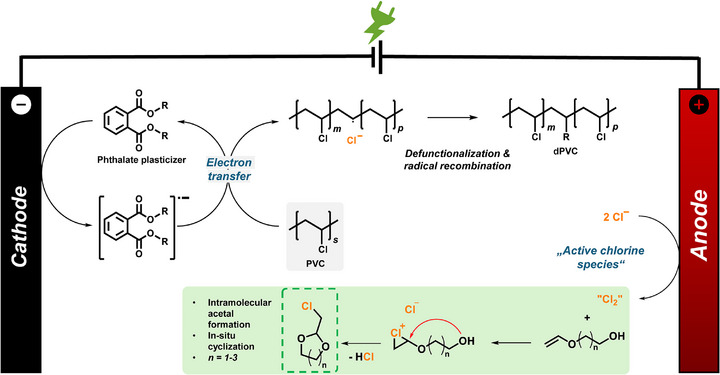
Chlorine shuttle (proposed mechanism) with paired electrolysis pathway: Reductive cleavage of the carbon‐chlorine bond (cathode) mediated by alkyl phthalates. Generation of active chlorine species (anode) leading to double bond chlorination and in situ cyclization of the vinyl ether to the cyclic acetal.

The electrochemical chlorination of vinyl ethers using Lindane as a chlorine source has previously been reported by waldvogel and coworkers.^[^ ^48^ ^]^ The electrochemical chlorination of vinyl ethers bearing an electron‐rich double bond, like ethyl vinyl ether, yields the corresponding dichloro product only in trace amounts. The main product observed is the acetal 2‐chloro‐1,1‐di(ethoxy)ethane. The presence of a terminal hydroxyl group enables intramolecular ring formation, yielding cyclic acetals. Combining this oxidative chlorination reaction at the anode with the electrochemical reductive dechlorination of PVC at the cathode represents an innovative paired electrolysis for PVC recycling.^[^ ^60^ ^]^ mcneil and coworkers have reported the selective chlorination of arenes while the electron deficient aromatic plasticizer di(2‐ethylhexyl) phthalate is not chlorinated.^[^ ^31^ ^]^ Here, we hypothesize that the electron‐rich double bond of vinyl ethers enables similar selectivity during electrolysis and subsequently enables efficient acetal formation via intramolecular cyclisation. **Figure** **1** depicts the proposed mechanism of the paired electrolysis pathway. The phthalate plasticizer enables electron transfer from the cathode onto PVC, initiating the cleavage of the C─Cl bond.^[^ ^31^ ^]^ Since the second reduction of the polymer radical is emphasized to proceed faster than the reductive C─Cl cleavage, a subsequent immediate second reduction and protonation lead to defunctionalized PVC (dPVC).^[^ ^61^, ^62^ ^]^ Another possible pathway is the radical recombination of polymer backbone radicals. Besides the recombination with other polymer radicals, phthalate esters are known to be electrochemically unstable and degrade into fragments.^[^ ^63^ ^]^ These species can also recombine with the polymer backbone.

**Figure 2:**
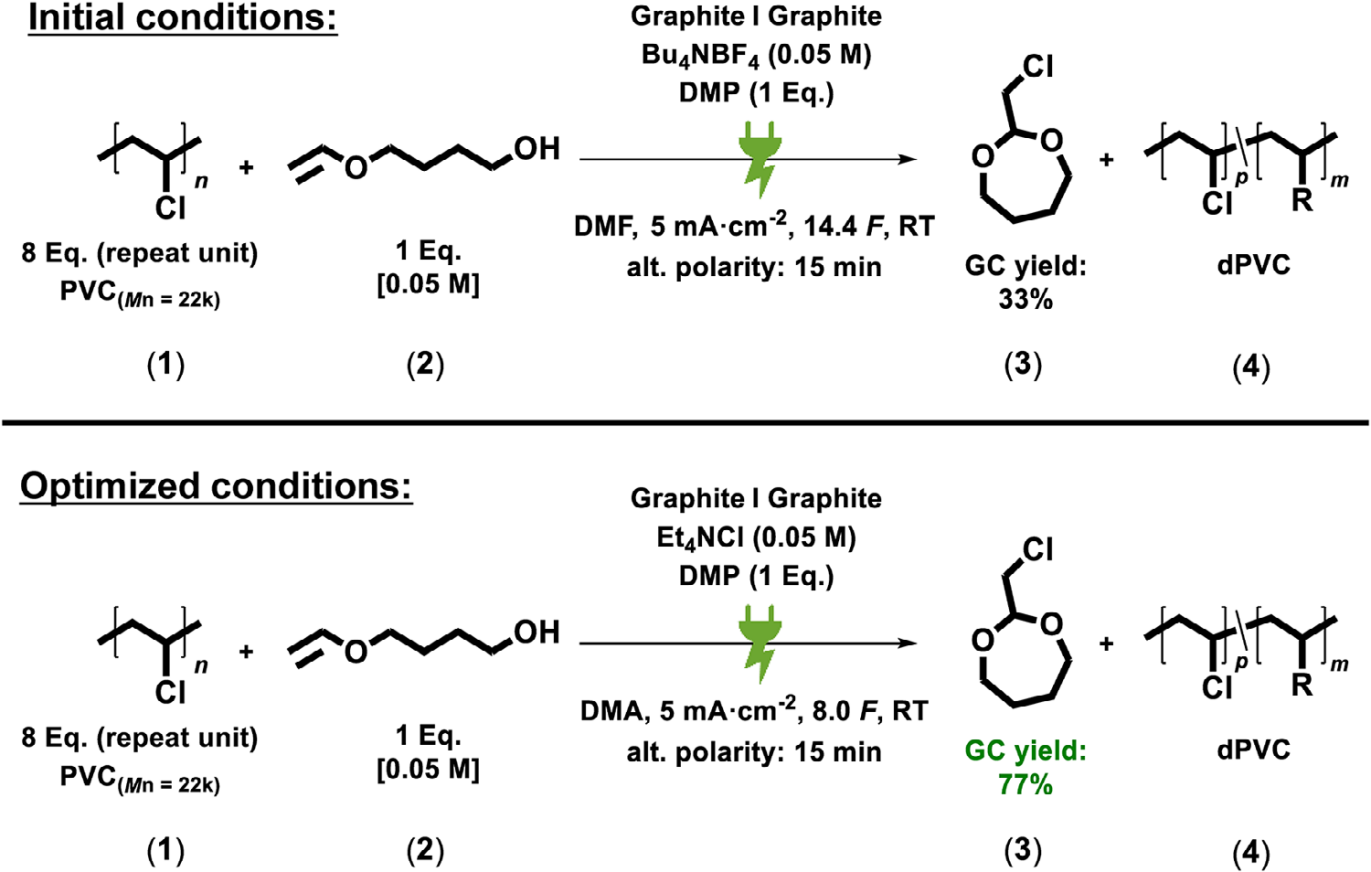
Initial conditions: Electrochemical reaction conditions used for optimization. Optimized conditions were obtained after electrochemical parameter screening was performed.

Electrochemical screening was started with low molecular weight PVC (*M*_n_ = 22.000 g^.^mol^−1^), and dimethyl phthalate (DMP) was chosen to simplify analysis of the polymers via nuclear magnetic resonance (NMR) spectroscopy. Starting with the initial conditions, we performed systematic screening, which led to the optimized conditions yielding 77% of the cyclic acetal **3** according to gas chromatography (GC) analysis (**Figure** **2**; Tables S2–S12, Supporting Information). Key parameters investigated were the solvent and supporting electrolyte, using *N,N*‐dimethylacetamide (DMA) in combination with alkylammonium chloride salts to significantly increase the GC yield of the cyclic acetal. Due to the use of chloride salts as supporting electrolytes, control experiments were performed to confirm that PVC dechlorination is essential for cyclic acetal formation. Electrolysis in the absence of PVC resulted in a substantially lower product yield of **3** (8%). Besides optimizing the yield of the cyclic acetal, our main focus was to avoid polymer network formation via radical recombination. To this end, slow alternating polarity was applied to prevent cathode corrosion or passivation caused by crosslinked polymer material. Screening results of the alternating polarity frequency (Table S12, Supporting Information), together with the trend that longer alkyl chain lengths of the ammonium cations led to higher GC yields of (**3**) suggested that an intact electrochemical double layer with an apolar microenvironment is crucial for the generation of active chlorine species and subsequent cyclic acetal formation.^[^ ^64^, ^65^ ^]^ Consequently, a compromise between maximizing cyclic acetal formation and minimizing network formation was necessary. Such trade‐offs are characteristic of paired electrolysis methods.^[^ ^60^ ^]^ Applying the optimized conditions resulted in high cyclic acetal yields of 77%, and GC‐MS analysis confirmed the absence of side reactions such as recombination of (**2**) to dimers (Figure S2, Supporting Information).

### 2.2 Increasing the Dechlorination Rate of PVC by Design of Experiments Approach

Having the optimized conditions in hand, further effort was invested toward increasing the dechlorination rate of PVC via electrochemical reduction. An eight‐fold excess of the PVC repeating units relative to the chlorine acceptor, 4‐hydroxybutyl vinyl ether (**2**), was applied in the optimization screening. In order to increase polymer dechlorination and subsequent Cl‐shuttle to the vinyl ether, the PVC excess was reduced to a 5:1 ratio, which led to a decreased GC yield of 63% regarding the cyclic acetal formation. This experiment served as a starting point for the optimization via Design of Experiment (DoE) approach.^[^ ^66^, ^67^, ^68^ ^]^

**Figure 3:**
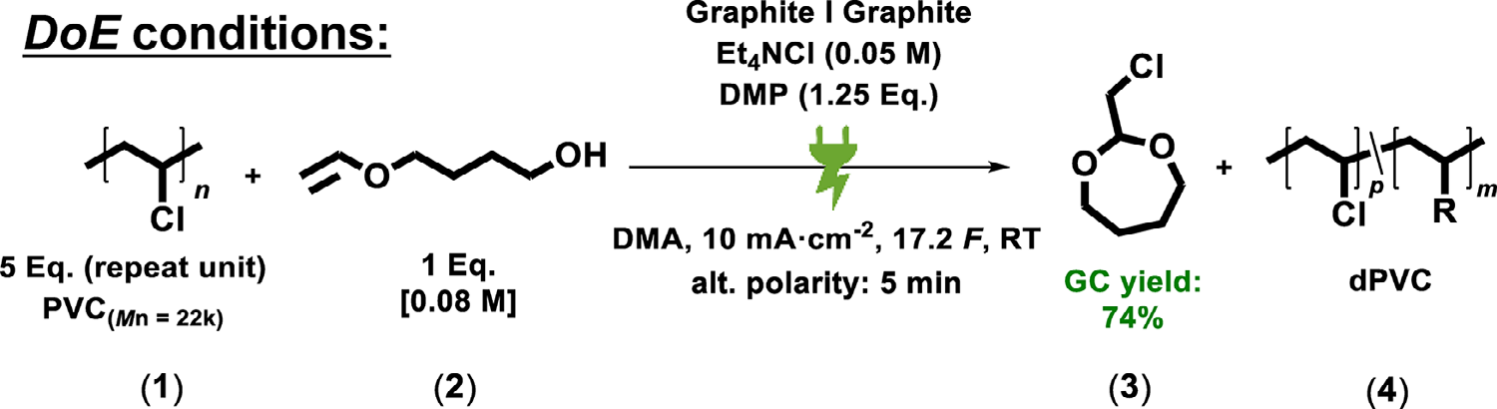
DoE conditions with decreased excess PVC (**1**) to enhance the dechlorination rate of the polymer material.

For the DoE screening plan, parameters such as current density, amount of applied charge, and phthalate concentration were selected (Table S13, Supporting Information). Having polymers involved, previous DoE screenings taught that the choice of screening parameters with reasonable framework conditions is crucial for successful optimization. For example, the screening of the polymer concentrations or polymer/chlorine acceptor ratio results in significant viscosity changes of the electrolyte, influencing mass transport and convection. This effect introduces considerable amounts of noise into the statistical model and reduces reproducibility, leading to a low R^2^ *_corr_* value.^[^ ^67^ ^]^ The subsequent addition of star points and target value optimization led to adjusted values for the screened parameters that increased the obtained GC yield of the cyclic acetal from an initial 63% to 74% (control experiment without PVC: 9%). **Figure** **3** illustrates the conditions that were obtained via DoE screening. Compared to the optimized conditions, the DoE‐derived adjustment of the selected parameters is suggested to result in a significantly increased dechlorination rate due to the lower excess of PVC.

DoE screening provided access to interaction plots of the selected parameters (current density, amount of applied charge, and phthalate concentration).^[^ ^66^ ^]^ These interaction plots (Table S14, Supporting Information) reveal that the phthalate concentration influences the optimal current density and optimal amount of applied charge for efficient cyclic acetal formation. Notably, this result could be particularly valuable for the recycling of real PVC products, which intrinsically contain certain phthalate loadings in their material formulation. Considering the electrochemical recycling of various PVC waste stream batches, the phthalate concentration could be determined, and subsequently, the electrochemical parameters (e.g., current density and applied charge) could easily and rapidly be adjusted to ensure optimal electrolysis performance for cyclic acetal formation.

**Figure 4:**
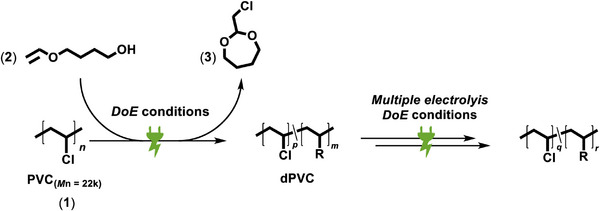
Multiple electrolysis reactions are applied to the same PVC (**1**) material to enhance the rate of dechlorination induced by electrochemical reduction.

In order to maximize the dechlorination of PVC using electrochemical reductive pathways, the DoE conditions were applied repeatedly for the same PVC material (**Figure** **4**). After each electrolysis, the material was precipitated, dried, and subsequently redissolved in DMA for reuse in the next electrolysis cycle. The GC yield of the cyclic acetal decreased stepwise from 74% in the first electrolysis to 54% in the second, 32% in the third, and 17% in the fourth, gradually approaching the value of the negative control (without PVC, 9%). Due to the coiled nature of the PVC material in solution, the inhomogeneous accessibility of C─Cl sites and the decreasing excess of PVC repeat units over the multiple electrolysis cycles influence and limit the cyclic acetal formation. Nevertheless, the experiment strongly suggests that high dechlorination rates are obtained.

### 2.3 Polymer Characterization

**Figure 5:**
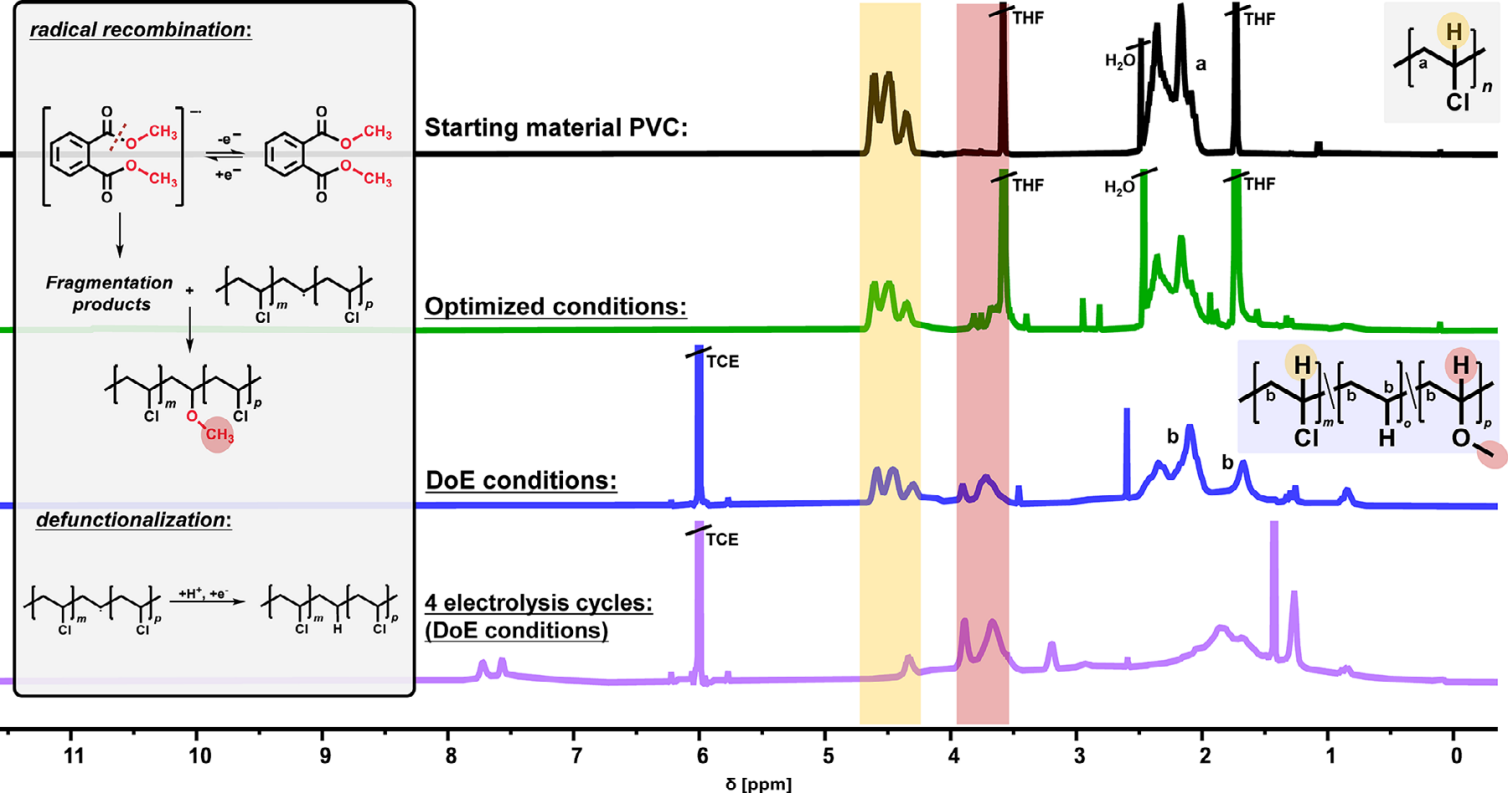
^1^H NMR analysis of the starting material (*black*, 400 MHz, THF‐*d*_8_, 298 K) and the dechlorinated PVC obtained from the optimized conditions (*green*, 400 MHz, THF‐*d*_8_, 298 K), DoE conditions (*blue*, 400 MHz, TCE‐*d*_2_, 298 K) and multiple electrolytic cycles (*violet*, 400 MHz, TCE‐*d*_2_, 298 K) using the DoE conditions. The decreasing intensity in the area between *δ* = 4.2–4.7 ppm (yellow area) indicates a gradual increase of the dechlorination rate. Recombination reactions with phthalate ester fragments are observable in between *δ* = 3.6–3.9 ppm (red area) with proposed mechanisms for the defunctionalization and radical recombination reaction.

The dechlorinated PVC was analyzed upon electrolysis by NMR spectroscopy and size exclusion chromatography (SEC). Additionally, thermogravimetric analysis (TGA) and elemental analysis were performed to gain deeper insight into the extent of PVC dechlorination. First, ^1^H NMR analysis (**Figure** **5**) of PVC samples obtained after electrolysis under optimized and DoE conditions revealed a stepwise decrease in the relative intensity of the broad ^1^H NMR signals for the R_2_CHCl group in the range of 4.2–4.7 ppm, confirming dechlorination of PVC. In addition, the broadening of the ^1^H NMR signals in the aliphatic region and the emerging signals between 3.6–3.9 ppm indicate defunctionalization and incorporation of methoxy groups, due to phthalate methyl ester fragmentation with subsequent radical recombination.^[^ ^63^ ^]^ mcneil and coworkers not only observed recombination with methoxy groups for higher dechlorination rates, but also additional ^1^H NMR signals in the aromatic region and a significant amount of double bond formation during electrochemical dechlorination with dimethyl phthalate. In contrast, our optimized and DoE electrolysis conditions in this work exclusively led to recombination with methoxy groups.^[^ ^31^ ^]^ Interestingly, the incorporation of the fragmentated aromatic phthalate moiety and double bond formation appear to be suppressed under these conditions.

**Figure 6:**
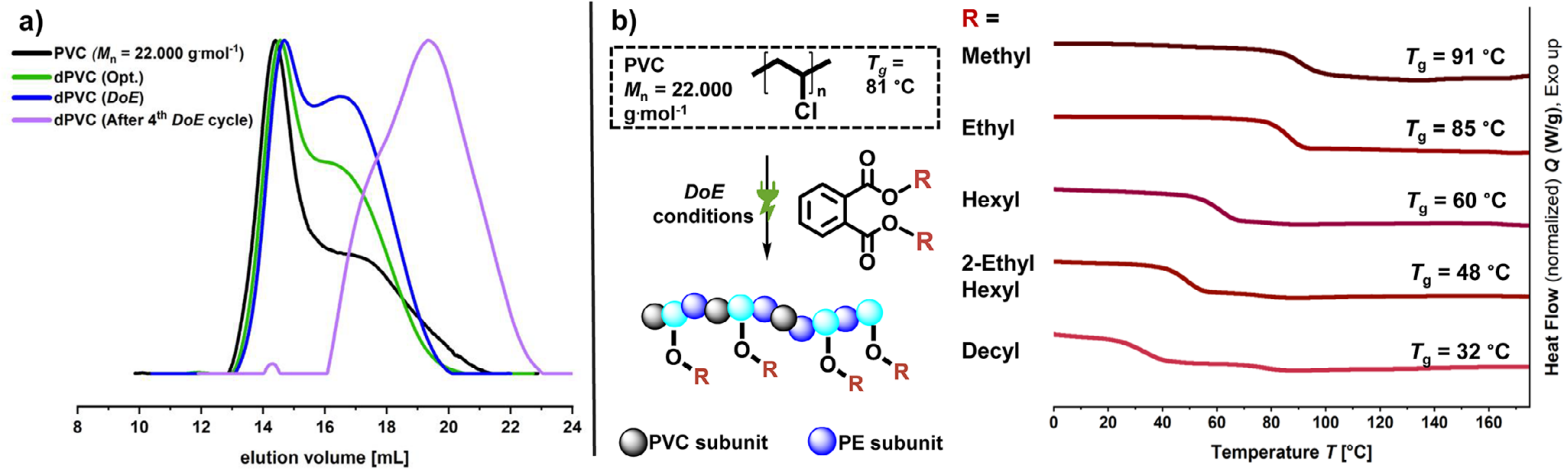
a) SEC elution traces of the starting material (*black*) and the dechlorinated PVC after electrolysis obtained from the optimized conditions (*green*), DoE conditions (*blue*) and multiple electrolytic cycles (*violet*). RI signal; eluent: DMF, 40 °C; standard: PMMA. b) Five different phthalate esters employed during electrolysis and their impact on the thermal properties of the produced dechlorinated PVC polymer materials.

The observed dechlorination of PVC was confirmed by TGA analysis (Figure S3, Supporting Information). Elemental analysis (Table S16, Supporting Information) further revealed a dechlorination rate of 25% under optimized conditions and 36% under DoE conditions, corresponding to a reduction of the chlorine content of the starting material from 56 to 42 and 36 wt.%, respectively. After four electrolytic cycles under DoE conditions, the previously discussed trend is furthermore confirmed: The characteristic PVC signal in the 4.2–4.7 ppm area is not detectable anymore, indicating that the original polymer backbone has been almost completely dechlorinated. Methoxy recombination accumulates over the four electrolysis cycles and, in contrast to the other polymer samples, additional ^1^H NMR signals indicative of recombination with aromatic phthalate groups become apparent. Notably, elemental analysis revealed chlorine content as low as 3.6 wt.% and corresponds to a 94% dechlorination rate. Although radical recombination involving aromatic and methoxy groups distorts the elemental composition, the results suggest close to quantitative dechlorination reactions. The potential and efficiency of the electrochemical modification of the PVC polymer backbone is remarkable, especially given the harsh conditions typically required to cleave high‐energy bonds such as the carbon–chlorine (C–Cl) bond. Size exclusion chromatography was furthermore employed and reveals a shift toward lower apparent molecular weights for all samples (**Figure** **6**a). We attribute this observation to the mass loss during dechlorination and altered hydrodynamic properties of the defunctionalized PVC in *N*,*N*‐dimethylformamide (DMF). Importantly, the absence of high molecular weight species confirms that no recombination reactions of polymer backbone radicals occurred.

The established electrochemical reaction under optimized or DoE conditions can be readily performed and reproduced using a variety of phthalate esters, such as di(2‐ethylhexyl) phthalate (DEHP). Functionalization of the polymer material with alkoxy side chains derived from fragmented phthalate esters, in combination with partial defunctionalization and conservation of the macromolecular architecture, results in an internally plasticized, partially dechlorinated PVC species. This material could be of interest for advanced upcycling strategies.^[^ ^69^, ^70^, ^71^, ^72^ ^]^ Differential scanning calorimetry (DSC) experiments and measurements of the glass transition temperatures (*T*_g_) were therefore performed of the dechlorinated PVC after employing phthalate esters with increasing aliphatic chain lengths in the electrochemical reaction (Figure 6b). While the recombination of dimethyl and diethyl phthalate fragments leads to an increase in *T*_g_ compared to PVC (anti‐plasticization), using industrially relevant phthalate esters such as DEHP in electrolysis cause a stepwise reduction in *T*_g_ with increasing alkyl chain length, reaching 32 °C for the didecyl phthalate. Analysis by ^1^H NMR spectroscopy revealed a further lowering in chlorine content in the dechlorinated PVC when using DEHP or didecyl phthalate during electrolysis, yielding chlorine weight percentages of 29 and 30 wt.%, respectively. This decrease is attributed to the incorporation of longer alkoxy side chains from these plasticizers, which alters the elemental composition of the resulting material. In comparison, using dimethyl phthalate in electrolysis resulted in a chlorine content of 36 wt.% as already discussed, since the shorter alkyl chains have a smaller impact on the overall elemental balance.

Internal PVC plasticization prevents embrittlement and avoids migration of toxic plasticizers, as no diffusion‐based leaching can occur. This is usually realized via polymer modification by nucleophilic substitution (S_N_) reactions using amines, azides, or thiols, graft polymerizations, or copolymerization.^[^ ^69^, ^70^, ^71^, ^73^, ^74^, ^75^, ^76^ ^]^ However, most approaches are not realizable on industrial scales due to high costs and the requirement of at least one, often several, additional synthetic steps.^[^ ^73^ ^]^ In our case, the internal plasticized material through this electrochemical process, yielding *T*_g_ values between 32–60 °C, could already find application of semi‐rigid PVC products, such as in flooring, trays, and blister packaging.^[^ ^76^ ^]^ This highlights the utility of the established paired electrolysis process not only for cyclic acetal synthesis as a precursor for polyesters, but also for generating partially dechlorinated, flexible materials with tunable mechanical properties and lower health risks. The process may be further optimized by adjusting electrochemical parameters and the amount of phthalate esters used during electrolysis, enabling the design of upcycled PVC materials with targeted thermal properties, which is a central aim of future investigations.

### 2.4 Process Stability

To evaluate the robustness, scalability, and applicability to real PVC waste streams, several additional experiments were conducted. Upscaling the electrochemical reaction to a 10 mL cell using DoE conditions showed no decrease in GC yield obtained for the cyclic acetal (**3**). A 50‐fold scale‐up using a 500 mL cell (250 mL reaction volume, 6.25 g PVC, 2.33 g (**2**)) resulted in a reduced yield of 35%, likely due to different cell architecture and stirring efficiency, indicating that while upscaling is feasible, further optimization for the corresponding cell size and reactor geometry is necessary. As expected, using high‐molecular‐weight PVC (*M*_n_ = 99.000 g^.^mol^−1^) led to slightly reduced yields: 64% under optimized conditions and 58% under DoE conditions. This can be attributed to two factors. First, a lower average accessibility of the carbon–chlorine sites, making dechlorination more challenging, and, second, increased viscosity which decreases mass transport during electrolysis. Rheological measurements showed how the different molecular weight PVC species (22.000 and 99.000 g mol^−1^) influence the viscosity of the electrolyte solution (Figure S5, Supporting Information). Control experiments without stirring, carried out under optimized conditions in a 5 mL screening cell, showed a lower GC yield of 32% (down from 77%), highlighting the critical importance of mass transport and consistent stirring during electrolysis and offering a rationale for the decreased yields observed in scale‐up and high molecular weight PVC experiments.

To further assess the robustness of the electrochemical reaction, a range of hard and soft PVC products were collected, cut into small pieces, and dissolved in DMA for electrolysis. NMR analysis confirmed the presence of PVC along with typical additives such as plasticizers, stabilizers, and minor impurities (Figure S6, Supporting Information). Some samples, such as polymer blends containing PE films (e.g., credit card coatings), resulted in suspensions that were subjected to electrolysis without filtration. The resulting GC yields of cyclic acetal (**3**) are summarized in **Table** **1**, and the results demonstrate the applicability of this electrochemical method to real PVC waste streams, including commercial additives, polymer blends, and non‐PVC formulation impurities.

**Table 1:** Use of PVC waste and consumer goods for the synthesis of 2‐chloromethyl‐1,3‐dioxepane (**3**). Optimized (Figure 2) and DoE conditions (Figure 3) were applied.

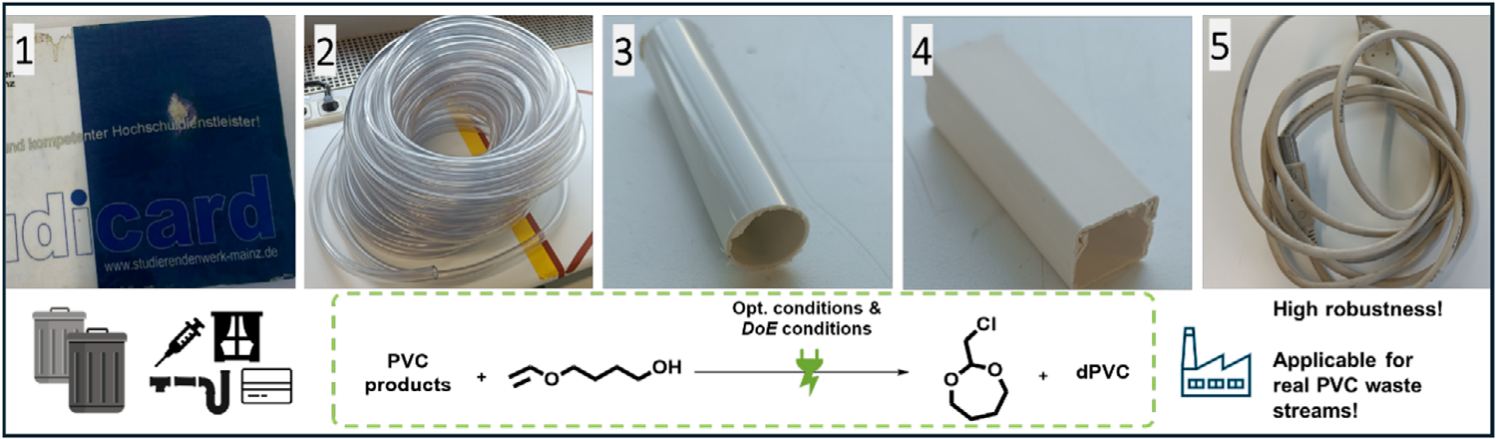		
PVC material	GC yield (2‐chloromethyl‐1,3‐dioxepane (3))/%	
	Optimized Cond.	DoE‐Conditions
PVC **(*M*_n_ = 99.000 g^.^mol^−1^)**	64	58
1‐credit card	71	43
2‐water hose	72	63
3‐tube	68	56
4−cable casing	59	70
5‐cable sheathing	65	51

Additionally, the PVC materials that exhibited the lowest GC yields under the DoE conditions, material‐1 (credit card, 43%) and material‐5 (cable sheathing, 51%), as listed in Table 1, were selected for additional experiments, in order to verify the conclusions obtained from the DoE screening described in section 2.2. NMR and GC‐MS analysis of material‐5 (cable sheathing) revealed high additive loadings and characteristic phthalate signals in the ^1^H NMR spectrum and mass spectra (di‐*iso*‐nonyl phthalate). Consequently, the PVC to chlorine acceptor ratio shifted from 5:1 to a much lower, unknown value. As the DoE prescreening had shown that a further decrease in this ratio leads to a significant loss in yield, an additional estimated amount of 20 wt.% PVC was used in the electrochemical reaction. This adjustment increased the obtained GC yield of (**3**) from 51% to 65%. For the credit card sample, analytical data showed no presence of phthalate esters; however, mass spectrometry indicated the abundance of UV absorbers (benzotriazole UV‐326). As the phthalate concentration is lower compared to other PVC products, the interaction plots suggested a lower optimal current density for the electrolysis of this unplasticized, hard PVC. Reducing the current density from 10 to 6 mA cm^−2^ increased the GC yield from 43% to 64%, thereby underlining and confirming the key conclusions from the parameter interaction study by the DoE approach of the electrochemical reaction.

### 2.5 Scope of Cyclic Acetals

**Figure 7:**
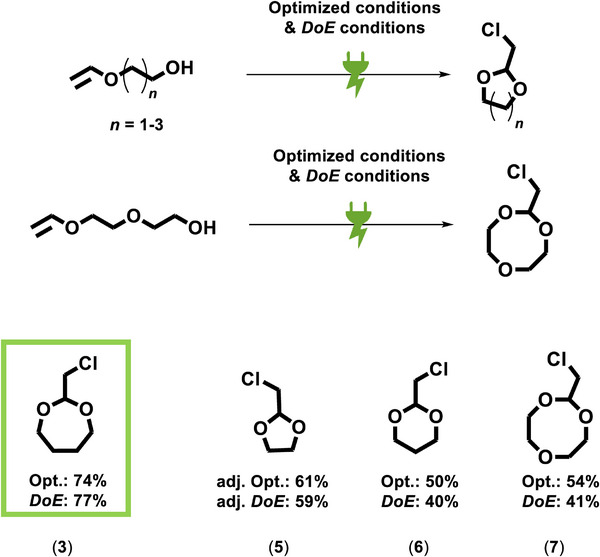
Scope of cyclic acetals, the electrochemical reaction conditions developed for efficient acetal formation of the seven‐membered ring applied to different sizes. Obtained GC yields using 1,10‐dichlorodecane as internal standard.

The reported electrochemical conditions for the formation of the seven‐membered acetal moiety (**3**) were applied to other vinyl ethers, altering the alkyl chain between the ether and the terminal hydroxy group. As a result, cyclic acetals with different ring sizes from five‐membered (**5**) to eight‐membered rings (**7**) were obtained (**Figure** **7**). The electrochemical reaction with intramolecular in situ cyclisation proceeded successfully, and the corresponding product formation was observed for all ring sizes, yielding the cyclic acetals (**5**)–(**7**) in moderate GC yields. The following basic elimination that provides the ketene acetal monomer is well documented in literature and proceeds in high yields.^[^ ^77^ ^]^ In this body of work, the established electrochemical reaction has been optimized for the seven‐membered cyclic acetal (**3**), since the corresponding ketene acetal shows superior polymerization features compared to the five‐ and six–membered cyclic ketene acetals, which are prone to side reactions during radical polymerization.^[^ ^55^ ^]^ The reactivity during polymerization is highly dependent on ring size (ring strain) and steric hindrance.^[^ ^56^ ^]^ To optimize the yield of the cyclic acetal 2‐chloromethyl‐1,3‐dioxolane (**5**), we adjusted the electrolysis conditions by using lower current densities and a lower supporting electrolyte concentration. Note that besides the seven‐membered cyclic ketene acetal, the eight‐membered ketene acetal also undergoes radical ring‐opening under a broad range of experimental conditions for homopolymer and copolymer synthesis.^[^ ^56^ ^]^ In particular, the eight‐membered ring (**7**) incorporates an additional oxygen atom, forming a diethylene glycol subunit within the resulting polyester repeat unit, which enhances water solubility. Hence, this substrate scope of cyclic acetals highlights the potential of our approach for providing easy access to a versatile platform of ketene acetal monomers that enable the design of novel, biodegradable materials.^[^ ^54^, ^56^, ^57^, ^59^, ^78^ ^]^

## 3 Conclusion

We have established a robust electrochemical paired electrolysis strategy for the dechlorination of PVC, enabling shuttle of chlorine equivalents from PVC to organic acceptors (vinyl ethers) and the concurrent formation of cyclic acetals. This process avoids the use and generation of hazardous molecular chlorine, proceeds under mild conditions and electric energy from renewable resources can be utilized. It yields useful monomer precursors, thereby avoiding the shift of waste problems to other chlorinated organic compounds on an industrial scale. Guided by a Design of Experiments (DoE) screening, this approach achieves high acetal yields and dechlorination efficiencies of up to 94% over multiple electrolysis cycles—significantly surpassing all previously reported electrochemical PVC dechlorination methods. Notably, the process maintains excellent functional robustness across different PVC types and waste products, in contrast to most literature‐known catalytic methods, which suffer from poor tolerance to additives and impurities, or challenges regarding scalability. The resulting PVC material exhibits internal plasticization, giving an optimal starting point for further upcycling strategies that will be addressed in the future. These findings highlight the potential of electrochemical chlorine shuttle chemistry as an innovative route for PVC recycling and set the stage for a platform of cyclic acetals monomer precursors enabling advanced and versatile material design of biodegradable polyesters on industrial scales.

## 4 Experimental Section

### Electrolysis Equipment & Setup

Electrochemical reactions were performed in undivided cells (5 mL, IKA, ElectraSyn®) with constant current provided by a galvanostat power supply (Rohde & Schwarz R&S® HMP4040). For upscale experiments, undivided 10 mL (10 mL, IKA, ElectraSyn®) and 250 mL (Institute workshop) undivided cells were used. The isostatic graphite was cut by the Institute workshop into the needed dimensions (50 x 10 x 2 mm, for 5‐ and 10 mL cells). The caps for the electrochemical cells made of Teflon were milled by the institute's workshop to fit the cell thread and were equipped with suitable openings for the electrode holders. Polarization changers have been installed between the power supply and electrochemical cell to change polarity (direct current to alternating current) during electrolysis and were manufactured by the institute's electricity workshop. For the electrochemical screenings, the starting materials were weighed directly into the electrochemical cell (5 mL). After complete homogenization, the Teflon cap with the electrode holders and electrodes was screwed onto the electrochemical cell. After the determination of the immersion depth, the contact clips were attached to the electrode holders. The electrochemical cell was put into a screening block (made of stainless steel by the institute's workshop) located on top of a stirring plate. For the upscale experiments, Teflon caps, electrodes, and electrode holders were used also manufactured by the institute's workshop. The 10 mL cell has been purchased commercially; the 500 mL cell was customized by the university's glass workshop.

### Nuclear Magnetic Resonance Spectroscopy

All NMR measurements (^1^H, ^13^C, HSQC, HMBC, and COSY) were carried out using an AVANCE II 400 MHz spectrometer from Bruker (^1^H: 400 MHz, ^13^C: 101 MHz) (Rheinstetten, Germany). All measurements were carried out at room temperature in a deuterated solvent. The chemical shifts were reported in relation to the solvent peak as an internal standard in parts per million (ppm), and the coupling constants *J* were measured in Hertz (Hz). The splitting patterns were labeled as s, singlet; d, doublet; t, triplet; dd, doublet of doublet; q, quartet; m, multiplet. The spectra were analyzed using *Mestrenova* software from *Mestrelab Research*. The deuterated solvents used were tetrahydrofuran‐*d_8_*, tetrachloroethane‐*d_2_*, dichloromethane‐*d_2,_* and chloroform‐*d_1,_* which were purchased from *Deutero*. (Germany)

### Gas chromatography

For the yield determination of electrochemical reactions were measured using the gas chromatograph GC 2010 from *Shimadzu* with a quartz capillary column ZB‐5 from the company *Phenomenex*. The column has a length of 30 m with an inner diameter of 0.025 mm. The covalently bonded stationary phase had a film thickness of 0.25 µm. The standard method included the following system settings: Hydrogen with a flow rate of 40.0 mL min^−1^ was used as carrier gas. The injection temperature was 250 °C and the detection temperature 320 °C. The method with a total run time of 19.94 min was used with a starting temperature of 40 °C. The starting temperature was maintained for 1 min. and then heated to 110 °C at a rate of 10 °C min^−1^. The temperature was then increased by 32 °C min^−1^ to 300 °C and held for 6 min. For yield determinations, product signal calibrations were performed using 1,10‐dichlorodecane (t_R_ = 11.21 min) as an internal standard.

*Size exclusion chromatography* (SEC) measurements were performed in DMF on an Agilent 1100 Series chromatograph from Agilent Technologies, Inc. The HEMA column set has a 300/100/40 Å porosity, and the samples were detected by RI and UV detectors (254 nm). *N,N*‐Dimethylformamide with 1 g mL^−1^ lithium bromide at 50 °C served as the mobile phase with a flow rate of 1 mL min^−1^. Toluene (1 µL mL^−1^) was used as an internal reference. For calibration, PMMA standards from Polymer Standard Service GmbH were used.

*Differential scanning calorimetry* (DSC) measurements were carried out using a DSC 250 instrument (TA Instruments) equipped with an RCS 90 cooling unit. The system was calibrated with indium and *n*‐octane as standard reference materials. ≈4‐7 mg of each polymer sample was weighed into an aluminum pan, which was then sealed. All measurements were performed under a constant nitrogen stream to prevent oxidative degradation. To eliminate any prior thermal history, samples were initially heated to 190 °C at a rate of 10 °C·min^−1^. Subsequently, DSC scans were conducted over a temperature range of –20 to 190 °C with a constant heating rate of 20 °C·min^−1^. Each experiment included two heating cycles and one intermediate cooling cycle. The glass transition temperature (*T*_g_) was determined from the second heating run, based on the inflection point of the corresponding heat capacity change.

*Elemental analysis* was performed by micro analytical laboratory *Kolbe* in Oberhausen, Germany.

### Design of Experiments Software

Evaluation of the experiments and response optimization of the DoE experimental plan were performed using *Minitab* Statistical Software 22 (Minitab LLC).

### General Electrolysis Procedure

(5 mL cell size, Optimized conditions) The polymer material PVC (100 mg, 1.6 mmol, 8 eq.(repeat unit)), the supporting electrolyte tetraethylammonium chloride (33.1 mg, 0.2 mmol, 1 eq, 0.05 M), dimethyl phthalate (38.8 mg, 0.2 mmol, 1 eq.) and 1,4‐butanediol mono vinyl ether (23.2 mg, 25 µL, 0.2 mmol, 1 eq.) were transferred into the electrochemical cell (ElectraSyn® 5mL) and dissolved in *N*,*N*‐dimethylacetamide (4 mL). The reaction mixture was stirred (500 RPM) for 15 min at room temperature using a Teflon‐coated magnetic stir bar. The electrodes and the teflon cap were attached to the reaction vessel, and the electrolysis was started. (*Q *= 8 *F*, *j* = 5 mA∙cm^− 2^, alt. Polarity = 15 min (4 h^−1^), electrode surface area ≈3.0 cm^−2^). After the reaction was finished, the internal standard 1,10‐dichlorodecane (50 µL) was added and stirred for 5 min. For yield/conversion determination, samples for gas chromatography were prepared. For polymer analysis, the solution was added dropwise into methanol (50 mL) to induce polymer precipitation. After centrifugation, the obtained polymer was redissolved in THF (3 mL) and precipitated again in methanol. After centrifugation, the polymer was dried *in a vacuum* and obtained as an amorphous, colorless solid.

### Isolation of 2‐Chloromethyl‐1,3‐Dioxepane (**3**)

After the electrolysis was finished, the reaction solution was added dropwise into methanol. The precipitated polymer was centrifuged, and the solution was transferred into a round bottom flask. Methanol was removed under reduced pressure. Lithium chloride solution (5%) was added to the residual mixture, and the aqueous solution was extracted with diethyl ether. The organic phase was extracted with 5% lithium chloride solution and dried with MgSO_4_. After filtration, the solvent was removed *in vacuo* , and obtained the product via distillation at 82 °C/22 mbar. (Colorless liquid, isolated yield of 53%) ^1^H‐NMR (400 MHz, C*D*Cl_3_, 298K): *δ*/ppm = 4.85 (td, *J* = 5.2, 1.2 Hz, 1H, O‐C**H**‐O), 3.95 (m, 2H, O‐C**H**_2_), 3.77 (m, 2H, O‐C**H**_2_), 3.47 (dd, *J* = 5.3, 1.1 Hz, 2H, Cl‐C**H**_2_), 1.71 (m, 4H, C**H**_2_‐C**H**_2_). ^13^C‐NMR (101 MHz, C*D*Cl_3_, 298K): δ/ppm = 100.96 (O‐**C**H‐O), 66.76 (O‐**C**H_2_), 44.40 (**C**H‐Cl), 29.32 (O‐CH_2_‐**C**H_2_), GC‐MS (Std. method, column: Rxi‐5Sil MS): t_R_ = 5.67 min. GC (Std. method, column: ZB‐5): t_R_ = 7.90 min.

## Conflict of Interest

The authors declare no conflict of interest.

## Supporting information



Supporting Information

## Data Availability

The data that support the findings of this study are available in the supplementary material of this article.
