# Supplementary material for: Electrochemical Chlorine Shuttle from PVC Waste to Vinyl Ether Acceptors for the Synthesis of Biodegradable Polyester Precursors
Source: Adv Mater. 2025 Nov 12;38(6):e17489. doi: 10.1002/adma.202517489 (PMC12848649; doi:10.1002/adma.202517489)
Supplement: Supplementary file 1 — Supporting Information [file ADMA-38-e17489-s001.pdf]

ADVANCED MATERIALS

Supporting Information

for *Adv. Mater.*, DOI 10.1002/adma.202517489

Electrochemical Chlorine Shuttle from PVC Waste to Vinyl Ether Acceptors for the Synthesis of Biodegradable Polyester Precursors

Sebastian Becker, Dominik Wördehoff, Dominik Weis, Simon Horsinka, Siegfried R. Waldvogel and Pol Besenius**

Supplementary Information for

Electrochemical Chlorine Shuttle from PVC Waste to Vinyl Ether Acceptors for the Synthesis of Biodegradable Polyester Precursors

Sebastian Becker[1], Dominik Wördehoff[1], Dominik Weis[1], Simon Horsinka[1], Siegfried R. Waldvogel[2,3] and Pol Besenius[1]**

[1] Department of Chemistry, Johannes Gutenberg University Mainz, Duesbergweg 10–14, 55128 Mainz (Germany). besenius@uni-mainz.de

[2] Department of Electrosynthesis, Max-Planck-Institute for Chemical Energy Conversion, Stiftstraße 34–36, 45470 Mülheim an der Ruhr (Germany). siegfried.waldvogel@cec.mpg.de

[3] Institute of Biological and Chemical Systems−Functional Molecular Systems (IBCS−FMS), Karlsruhe Institute of Technology, Kaiserstraße 12, 76131 Karlsruhe (Germany).

Table of contents

GENERAL INFORMATION 3

SYNTHESIS OF 2-CHLOROMETHYL-1,3-DIOXEPANE (3) 6

ELECTROCHEMICAL SCREENING 8

INCREASING THE CHLORINE CLEAVAGE USING *DESIGN OF EXPERIMENTS* APPROACH 14

GENERAL PROCEDURES 17

POLYMER ANALYSIS 18

UPSCALE AND PVC (WASTE) PRODUCTS 22

CYCLIC ACETAL SCOPE 25

REFERENCES 30

SPECTRA 31

General Information

NMR Spectroscopy

All NMR measurements (^{1}H, ^{13}C, HSQC, HMBC and COSY) were carried out using an AVANCE II 400 MHz spectrometer from Bruker (^{1}H: 400 MHz, ^{13}C: 101 MHz) (Rheinstätten, Germany). All measurements were carried out at room temperature in deuterated solvent. The chemical shifts are reported in relation to the solvent peak as internal standard in parts per million (ppm) and the coupling constants *J* are measured in Hertz (Hz). The splitting patterns are labeled as s, singlet; d, doublet; t, triplet; dd, doublet of doublet; q, quartet; m, multiplet.

The spectra were analyzed using *Mestrenova* software from *Mestrelab Research*. The deuterated solvents used were Tetrahydrofuran-d_8, Tetrachloroethane-d_2, Dichloromethane-d_2 and Chloroform-d_1 that have been purchased from *Deutero*. (Germany)

Gas Chromatography (GC/GC-MS)

GC

GC-samples for yield determination of electrochemical reactions were measured using the gas chromatograph GC 2010 from *Shimadzu* with a quartz capillary column ZB-5 from the company *Phenomenex*. The column has a length of 30 m with an inner diameter of 0.025 mm. The covalently bonded stationary phase had a film thickness of 0.25 µm. The standard method included the following system settings: Hydrogen with a flow rate of 40.0 mL/min was used as carrier gas. The injection temperature was 250 °C and the detection temperature 320 °C. The method with a total run time of 19.94 min was used with a starting temperature of 40 °C. The starting temperature was maintained for one minute and then heated to 110 °C at a rate of 10 °C/min. The temperature was then increased by 32 °C/min to 300 °C and held for six minutes. For yield determinations product signal calibrations were performed using 1,10-dichlorodecan (t_R = 11.21 min) as internal standard.

GC/MS

Gas chromatographic measurements combined with mass spectroscopy were carried out on a *Clarus 600* gas chromatograph with an electron ionization detector from *Perkin-Elmer*. A quartz capillary column Rxi-5Sil MS from *RESTEK* (20 m long with an inner diameter of 0.18 mm) with a film thickness of 18 µm was used. Helium served as carrier gas and the sample injection volume was 1 µL. The standard method included the following temperature program with a total run time of 15 minutes: The starting temperature of 40 °C was maintained for 0.97 min. Then it was heated with a gradient of 13 °C/min to 85 °C and afterwards with a rate of 30 °C/min to 210 °C. This temperature was maintained for 6.40 min.

The chromatograms and the mass spectra were analyzed using *Mestrenova* software from *Mestrelab Research*.

Size exclusion chromatography (SEC/GPC)

GPC measurements were performed in DMF on an Agilent 1100 Series chromatograph from Agilent Technologies, Inc. The HEMA column set has a 300/100/40 Å porosity and the samples were detected by RI and UV detectors (254 nm). *N,N*-Dimethylformamide with 1 g/mL lithium bromide at 50 °C served as the mobile phase with a flow rate of 1 mL/min. Toluene (1 µL/mL) was used as internal

reference. For calibration Poly(methyl methacrylate) (PMMA) standards from Polymer Standard Service GmbH were used.

Thermo gravimetric analysis (TGA)

Thermogravimetric analysis (TGA) was conducted on a TGA 2 STAR^e system by *Mettler Toledo*. The samples (ca. 8 mg) were placed in crucibles made of polycrystalline aluminum oxide (PCA/Saphir). The samples were heated to 550 °C (10 °C/min) and the final temperature was kept for 60 minutes.

Elemental analysis

Elemental analysis was performed by micro analytical laboratory *Kolbe* in Oberhausen, Germany.

Differential scanning calorimetry

Differential scanning calorimetry (DSC) measurements were carried out using a DSC 250 instrument (TA Instruments) equipped with an RCS 90 cooling unit. The system was calibrated with indium and *n*-octane as standard reference materials. Approximately 8-10 mg of each polymer sample was weighed into an aluminum pan, which was then sealed. All measurements were performed under a constant nitrogen stream to prevent oxidative degradation.

To eliminate any prior thermal history, samples were initially heated to 120 °C at a rate of 10 °C min^{-1}. Subsequently, DSC scans were conducted over a temperature range of –90 °C to 120 °C with a constant heating rate of 10 °C·min^{-1}. Each experiment included two heating cycles and one intermediate cooling cycle. The glass transition temperature (T_g) was determined from the second heating run, based on the inflection point of the corresponding heat capacity change.

Design of Experiments Software

Evaluation of the experiments and response optimization of the DoE experimental plan were performed using *Minitab* Statistical Software 22 (Minitab LLC).

Electrolysis Equipment

All electrochemical reactions were performed in undivided cells (5 mL, *IKA*, ElectraSyn®) with constant current provided by a galvanostat (*Rohde & Schwarz R&S*® HMP4040). For upscale experiments undivided 10 mL (10 mL, *IKA*, ElectraSyn®) and 250 mL (Institute workshop) undivided cells were used. The isostatic graphite was cut by the Institute workshop into needed dimensions (50 x 10 x 2 mm, for 5- and 10 mL cell). The caps for the electrochemical cells made of Teflon were milled by the institute's workshop to fit the cell thread and were equipped with suitable openings for the electrode holders, which were made out of titanium to prevent corrosion. Polarization changers have been installed between power supply and electrochemical cell to change polarity (direct current to alternating current) during electrolysis and were manufactured by the institute’s electricity workshop.

Electrolysis Setup

For all electrochemical screenings the starting materials were weighed directly into the electrochemical cell (5 mL, IKA, ElectraSyn®). After complete homogenization the Teflon cap with the electrode holders and electrodes was screwed onto the electrochemical cell. After the determination of the immersion depth, the contact clips were attached to the electrode holders. The

electrochemical cell was put into a screening block (made of stainless steel by the institute's workshop) located on top of a stirring plate.

For the upscale experiments Teflon caps, electrodes and electrode holders were used also manufactured by the institute's workshop. The 10 mL cell has been purchased commercially; the 500 mL cell was customized by the universities glass workshop.

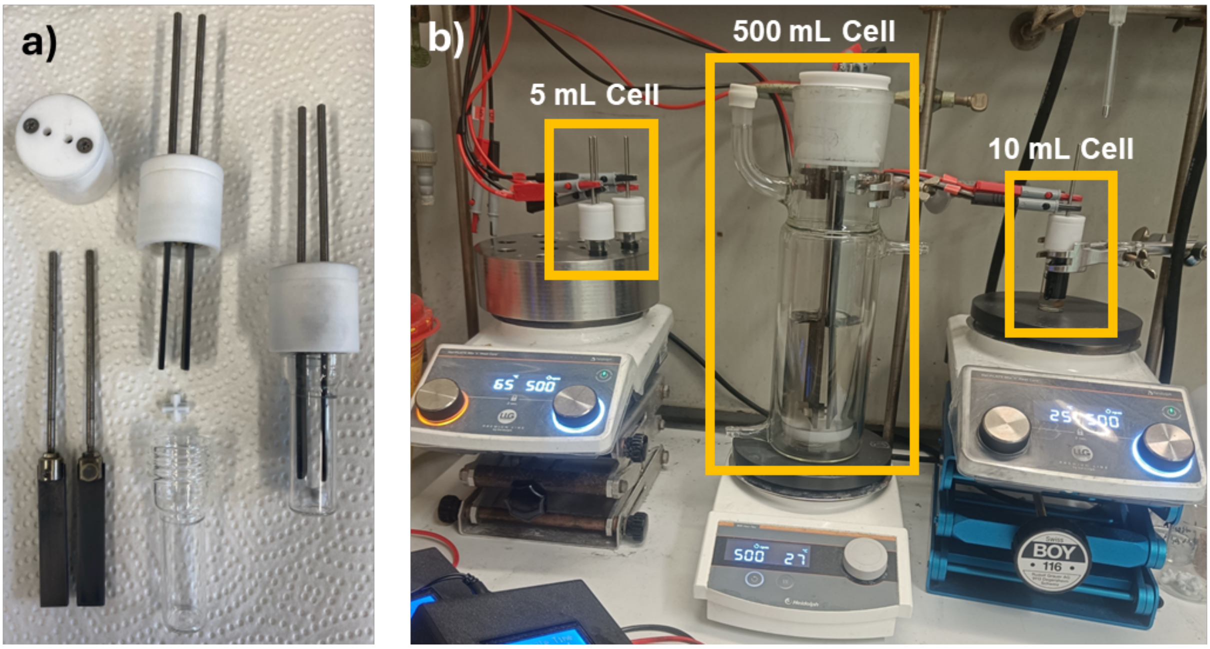

Figure S1: a) Components of the 5 mL cell for electrochemical screenings. b) reaction setup for electrochemical screening (left) and upscale experiments (500 mL cell (middle), 10 mL cell (right).

Synthesis of 2-Chloromethyl-1,3-dioxepane (3)

Classical synthesis was performed according to a protocol published from Tardy *et al.* After characterization, the obtained product was used for GC calibration and GC-MS identification.[1]

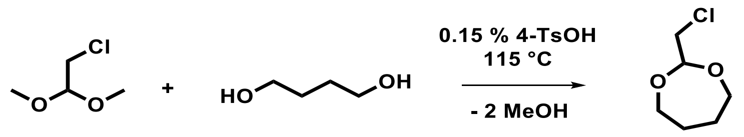

The synthesis was carried out in a distillation apparatus equipped with a stirring bar, a vigreux-column and cooling condenser. The following starting materials were introduced into the 250 mL round bottom flask: butane-1,4-diol (15.0 g, 166 mmol, 15.2 mL, 1 eq.), chloroacetaldehyde dimethylacetal (20.7 g, 166 mmol, 22.7 mL, 1 eq.) and 4-toluene sulfonic acid (0.04 g, 0.25 mmol, 0.002 eq.). Under constant stirring the solution was heated at 115 °C at a pressure of 700 mbar. After the produced methanol has been distilled (9.2 g obtained MeOH, theoretically 10.6 g of MeOH) the product was obtained as a colorless liquid at 95 °C/ 52 mbar applying a bath temperature of 139 °C. (22.6 g, 150 mmol, 90 % yield)

^{1}H-NMR (400 MHz, C*D*Cl$_3$, 298K): δ/ppm = 4.85 (td, J = 5.2, 1.2 Hz, 1H, O-C**H**-O), 3.95 (m, 2H, O-C**H**$_2$), 3.77 (m, 2H, O-C**H**$_2$), 3.47 (dd, J = 5.3, 1.1 Hz, 2H, Cl-C**H**$_2$), 1.71 (m, 4H, C**H**$_2$-C**H**$_2$).

^{13}C-NMR (101 MHz, C*D*Cl$_3$, 298K): δ/ppm = 100.96 (O-CH-O), 66.76 (O-**C**H$_2$), 44.40 (**C**H-Cl), 29.32 (O-CH$_2$-**C**H$_2$).

GC-MS (Std. method, column: Rxi-5Sil MS): t_R = 5.67 min.

GC (Std. method, column: ZB-5): t_R = 7.90 min.

GC-calibration plot for yield determination of 2-Chloromethyl-1,3-dioxepane (**3**)

Table S1: GC calibration for yield determination of 2-chloromethyl-1,3-dioxepane (3) using 1,10-dichlorodecane (1,10-DD) as internal standard.

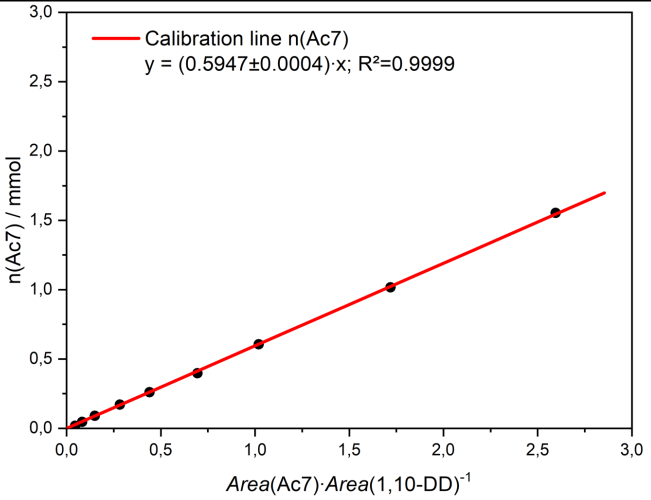

$A(3)\cdot A^{-1}$(1,10-dichlorodecane)	n((3)) / mmol
0.046	0.018
0.083	0.046
0.150	0.090
0.283	0.170
0.440	0.261
0.695	0.397
1.020	0.606
1.720	1.016
2.596	1.553

GC yield, 1,10-dichlorodecane as internal standard.

Electrochemical Screening

The starting conditions of the electrochemical screening are inspired by the electrochemical chlorination of arenes using PVC from McNeill *et al.*[2]

Initial conditions:

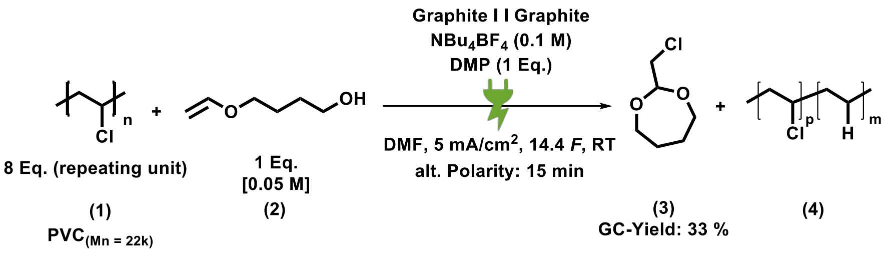

Starting from these electrochemical conditions the parameter screening was started by using the *OVAT (One variable at one time)*-screening approach. Major goal was increasing the obtained GC yield of the 2-chloromethyl-1,3-dioxepane (**3**), while avoiding crosslinking of PVC leading to precipitation and cathode passivation. The highlighted conditions in the tables (green line) have been chosen for following experiments.

<u>Screening of solvents</u>

Table S2: Solvent screening for the optimization of the electrochemical reaction. Yields of 2-chloromethyl-1,3-dioxepane (3) determined via gas chromatography using 1,10-dichlorodecane as internal standard.

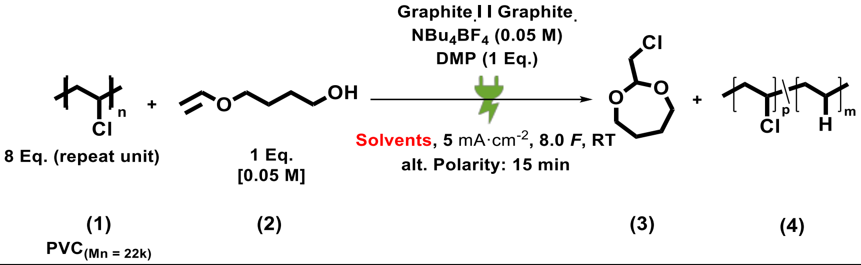

Solvent	GC yield (2-chloromethyl-1,3-dioxepane (**3**)) / %
DMF	33
NMP	16
Pyridine	0
Benzonitrile	0
TMU	13
DMA	47
Dioxane	0
DMF/Propylene carbonate 1:1 (v:v)	20
DMA/Propylene carbonate 1:1 (v:v)	30

GC yield, 1,10-dichlorodecane as internal standard.

Screening of supporting electrolytes

Table S3: Supporting electrolyte screening for the optimization of the electrochemical reaction. Yields of 2-chloromethyl-1,3-dioxepane (3) determined via gas chromatography using 1,10-dichlorodecane as internal standard.

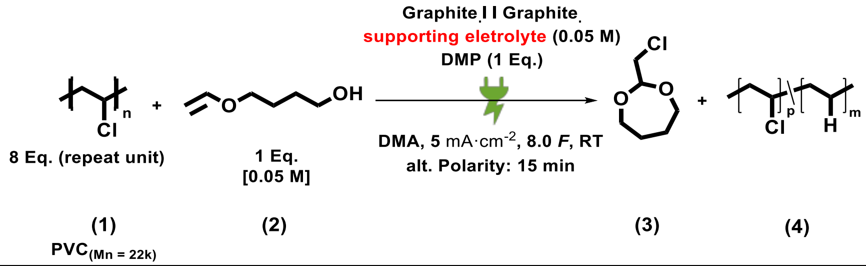

Supporting Electrolyte	GC yield (2-chloromethyl-1,3-dioxepane (**3**)) / %
Bu_4NI	12
Bu_4NBr	4
Et_4NPF_6	37
Bu_4NPF_6	44
Et_4NBF_4	44
Bu_4NBF_4	47
Bu_4NOTf	51
Et_4NHCO_3	34
Bu_4NOH (in H_20)	26
Bu_4NOH (in MeOH)	32
Bu_4NOAc	39
$MeEt_3NCl$	76
Et_4NCl	77
Bu_4NCl	79
Oct_3MeNCl	81

GC yield, 1,10-dichlorodecane as internal standard.

All ammonium chloride supporting electrolytes led to highly increased GC-yields of (**3**). The use of chloride containing supporting electrolytes required the execution of control experiments. (See next section) It was continued with the ammonium chloride salts, specifically due to its low costs with Et_4NCl to develop an economically attractive electrochemical reaction.

Screening of electrochemical mediators

Table S4: Screening of mediators for the optimization of the electrochemical reaction. Yields of 2-chloromethyl-1,3-dioxepane (3) determined via gas chromatography using 1,10-dichlorodecane as internal standard.

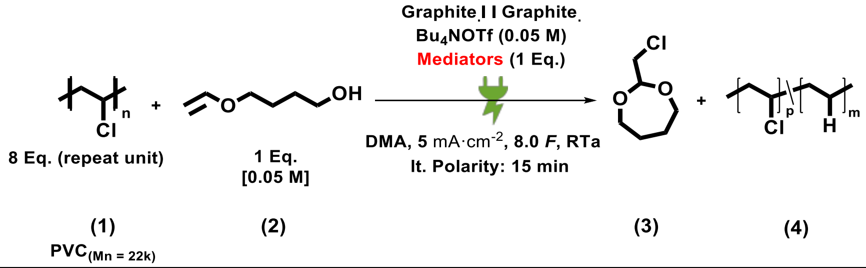

Mediator	GC yield (2-chloromethyl-1,3-dioxepane (**3**)) / %
Dimethylphthalate (DMP)	51
None	37
Benzonitrile	30
Phthalonitrile	51
4-Cyanopyridine	29
9-Phenylcarbazole	2
Dimethylterephtalate	36
$Mn(OTf)_2$	4
$Mn(OAc)_2$	35
$Ni(acac)_2$	35

GC yield, 1,10-dichlorodecane as internal standard.

Concentration screening

Screening of supporting electrolyte concentration

Table S5: Supporting electrolyte concentration screening for the optimization of the electrochemical reaction. Yields of 2-chloromethyl-1,3-dioxepane (3) determined via gas chromatography using 1,10-dichlorodecane as internal standard.

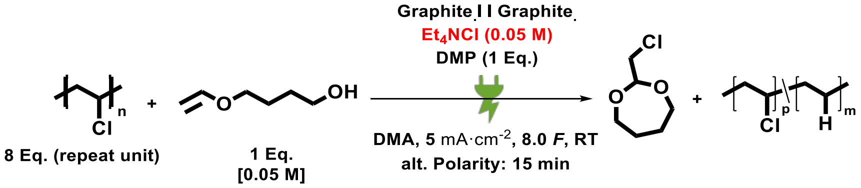

(1) PVC$_{(Mn = 22k)}$ (2) (3) (4)

$c(Et_4NCl)$ / mol L^{-1}	GC yield (2- hloromethyl-1,3-dioxepane **(3)**) / %
0.01	71
0.02	75
0.03	75
0.04	77
0.05	77

GC yield, 1,10-dichlorodecane as internal standard.

Screening of the mediator concentration

Table S6: Mediator concentration screening for the optimization of the electrochemical reaction. Yields of 2-chloromethyl-1,3-dioxepane (3) determined via gas chromatography using 1,10-dichlorodecane as internal standard.

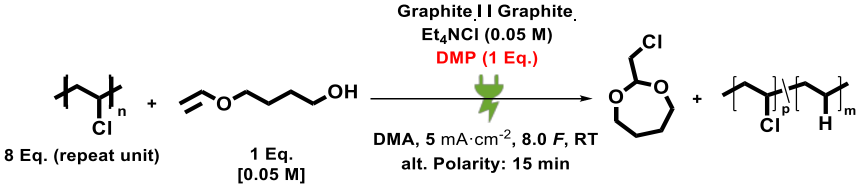

(1) PVC$_{(Mn = 22k)}$ (2) (3) (4)

c(DMP) / Eq.	GC yield (2-chloromethyl-1,3-dioxepane **(3)**) / %
None	34
0.5	40
1	66
1.5	66

GC yield, 1,10-dichlorodecane as internal standard.

Screening of the polymer concentrations

Table S7: Polymer concentration screening for the optimization of the electrochemical reaction. Yields of 2-chloromethyl-1,3-dioxepane (3) determined via gas chromatography using 1,10-dichlorodecane as internal standard.

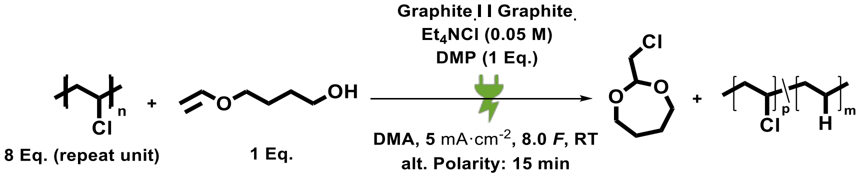

(1) PVC$_{(Mn = 22k)}$ (2) (3) (4)

Polymer concentration / mg mL^{-1}	GC yield (2-chloromethyl-1,3-dioxepane **(3)**) / %
25 (c(repeat unit) = 0.4 M)	77
37.5 (c(repeat unit) = 0.6 M)	62
50 (c(repeat unit) = 0.8 M)	52
62.5 (c(repeat unit) = 1.0 M)	46

GC yield, 1,10-dichlorodecane as internal standard.

Screening of PVC and chlorine acceptor ratio (excess of PVC)

Table S8: PVC (repeat unit) and chlorine acceptor (2) ratio screening for the optimization of the electrochemical reaction. Yields of 2-chloromethyl-1,3-dioxepane (3) determined via gas chromatography using 1,10-dichlorodecane as internal standard.

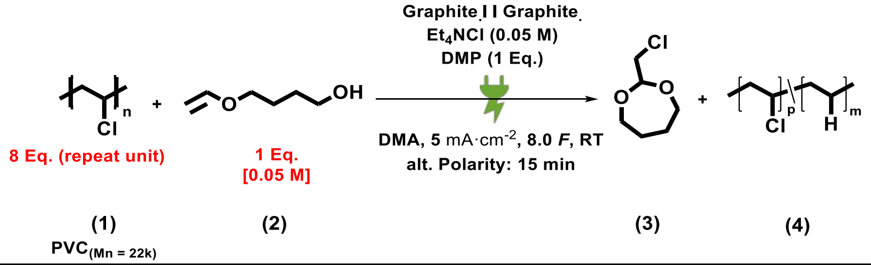

Eq. (PVC: chlorine acceptor (2))	GC yield (2-chloromethyl-1,3-dioxepane **(3)**) / %
(8:1)	77
(6.7:1)	63
(5.7:1)	64
(5:1)	63
(4.4:1)	47

GC yield, 1,10-dichlorodecane as internal standard.

Screening the influence of water

Table S9: Determining the influence of water for the optimization of the electrochemical reaction. GC yields of 2-chloromethyl-1,3-dioxepane (3) determined via gas chromatography using 1,10-dichlorodecane as internal standard.

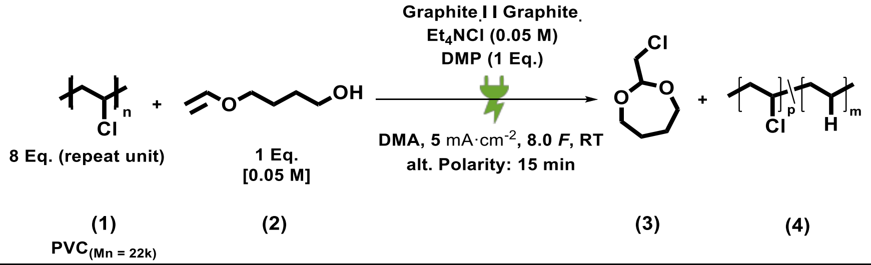

Added H_2O / Eq.	GC yield (2-chloromethyl-1,3-dioxepane **(3)**) / %
0 (Dry DMA, stored under Ar)	75
0	77
0.5	71
1	68
10	37
50	19

GC yield, 1,10-dichlorodecane as internal standard.

Screening of electrochemical, technical parameters

Screening the current density

Table S10: Screening of the current density for the optimization of the electrochemical reaction. GC yields of 2-chloromethyl-1,3-dioxepane (3) determined via gas chromatography using 1,10-dichlorodecane as internal standard.

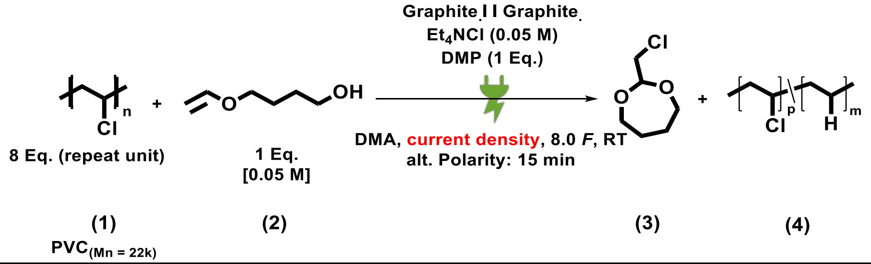

Current density / mA·cm^{-2}	GC yield (2-chloromethyl-1,3-dioxepane **(3)**) / %
2.5	50
5.0	77
7.5	73
10.0	55
12.5	34
15.0	42

GC yield, 1,10-dichlorodecane as internal standard.

Screening of the applied charge

Table S11: Screening of the applied charge for the optimization of the electrochemical reaction. GC yields of 2-chloromethyl-1,3-dioxepane (3) determined via gas chromatography using 1,10-dichlorodecane as internal standard.

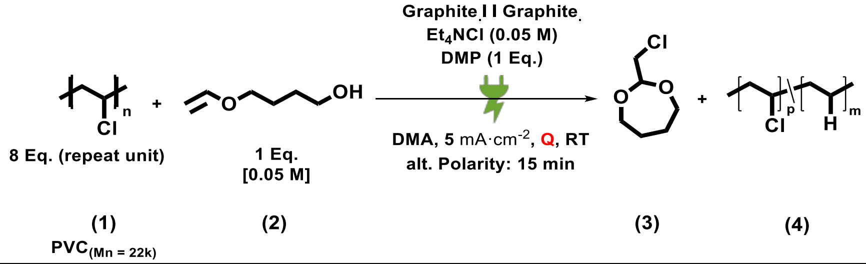

Applied charge Q / *F*.	GC yield (2-chloromethyl-1,3-dioxepane **(3)**) / %
0 (no current)	0
0.1	29
0.2	50
0.3	70
0.4	73
0.5	74
1.0	77
1.5	76
2.0	73
2.5	74

GC yield, 1,10-Dichlorodecane as internal standard.

Screening the alternating polarity frequency

Table S12: Screening of the alternating polarity frequency for the optimization of the electrochemical reaction. GC yields of 2-chloromethyl-1,3-dioxepane (3) determined via gas chromatography using 1,10-dichlorodecane as internal standard.

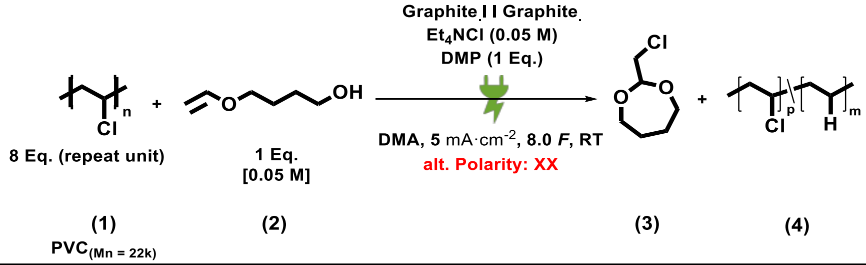

Alt. Polarity	GC yield (2-chloromethyl-1,3-dioxepane **(3)**) / %
3 s	7
15 s	65
30 s	71
3 min	74
30 min	75
Direct current	76

GC yield, 1,10-dichlorodecane as internal standard.

Optimized Conditions and Negative Controle

Optimized conditions: The electrochemical screening led to the following optimized conditions:

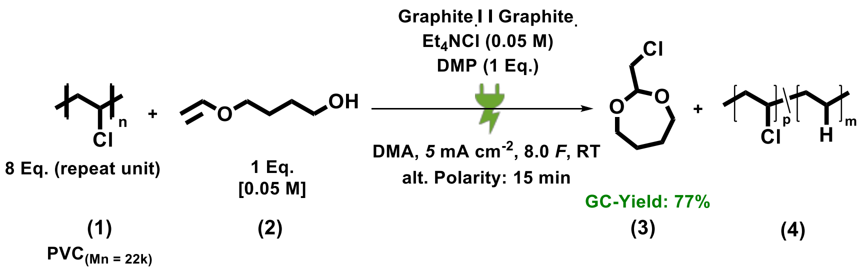

Besides the GC measurements for yield determination, GC-MS was performed. GC-MS showed full conversion of the starting material (**2**) and only the peaks of product (**3**), mediator (**DMP**) and the added internal standard. No side reactions have been observed.

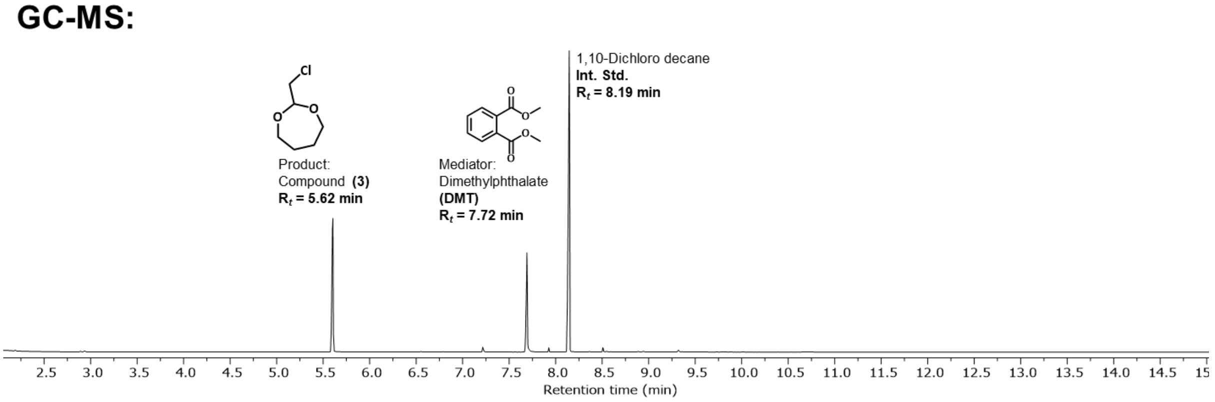

Figure S2: GC-MS chromatogram of the reaction solution after electrolysis.

Negative control: Due to the use of Et_4NCl as supporting electrolyte many control experiments have been performed (electrolysis reactions without PVC) during the electrochemical screening. The control experiments showed that PVC is essential for efficient product formation and only small amounts of chloride from the supporting electrolyte are involved in product formation. Also, the screening of supporting electrolytes concentration (Table S6) reveals that very low concentrations of Et_4NCl still lead to high GC yields of (**3**), proving that chlorine is cleaved electrochemically from PVC and used for product formation. Using the optimized conditions without PVC led to an obtained GC yield of 9%.

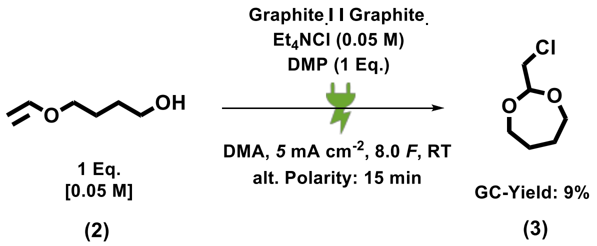

Increasing the Chlorine cleavage using *Design of Experiments* approach

To increase the dechlorination of the polymer material further the PVC (repeat unit): Chlorine acceptor ratio was changed to 5:1 (lower excess of PVC) leading to a decreased GC-yield of 63% for product (**3**). (Table 9)

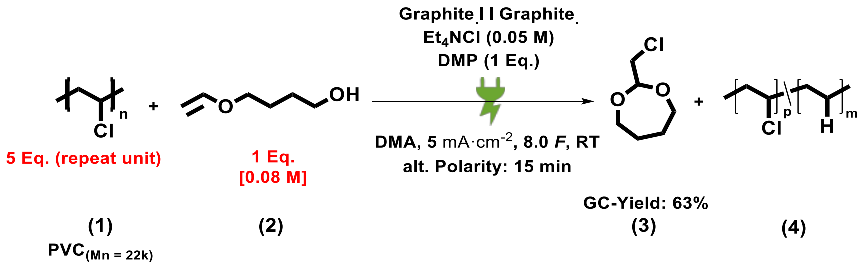

In the following, a *Design of Experiments* approach was chosen to study the influence of three parameters (current density, applied charge and mediator concentration) and their interactions in detail. A 2^3 full factorial experiment design was created, and certain parameter limits (current density *j*, amount of applied charge *Q*, and phthalate concentration c(DMT)) were chosen. (Table S14) The evaluation of the experiments was performed using *Minitab* Statistical Software 22 (Minitab LLC).

Table S13: Determined yields of 2-chloromethyl-1,3-dioxepane (3) in a 2^3 full factorial experimental with central composite design (CCD).

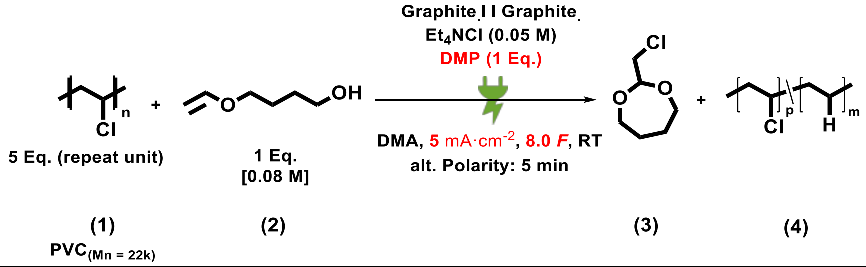

	TERM		target value
A	B	C	
Current densitiy<br>*j* / mA cm^2	Applied charge<br>Q / *F*	DMP (Eq.)	yield<br>(2-Chloromethyl-1,3-dioxepane (**3**)) / %
2.5	3.2	0.5	55 ± 0
2.5	12.8	0.5	57 ± 3
7.5	3.2	0.5	48 ± 2
7.5	12.8	0.5	38 ± 1
2.5	3.2	1.5	54 ± 2
2.5	12.8	1.5	61 ± 1
7.5	3.2	1.5	58 ± 1
7.5	12.8	1.5	64 ± 0
5.0	8.0	1.0	65 ± 1
5.0	0.8	1.0	16
5.0	17.6	1.0	65
0.5	8.0	1.0	41
10.0	8.0	1.0	62
5.0	8.0	0.1	39
5.0	8.0	2.0	63

GC yield: 1,10-dichlorodecane as internal standard.

The corner and central points of the 2^3 full factorial experiment design have been executed in duplicates. The standard deviation of these duplicates was chosen as error margin. The performed duplicates were well-reproducible. The statistical model exhibited an R^2_{corr} value of 92.9%.

Table S14: Main, Pareto and Interaction plots of the 2^2 full factorial experimental design for the product formation of 2-Chloromethyl-1,3-dioxepane **(3)**. In addition, the coded coefficients of the experimental design with the respective effects, coefficients (Coef), standard errors (SE Coef) of the coefficients, *t*- and *p*-value of the model (bottom). The calculation was based on a significance level of α = 5%.
Main effect A: current density
Main effect B: applied charge
Main effect C: phthalate concentration

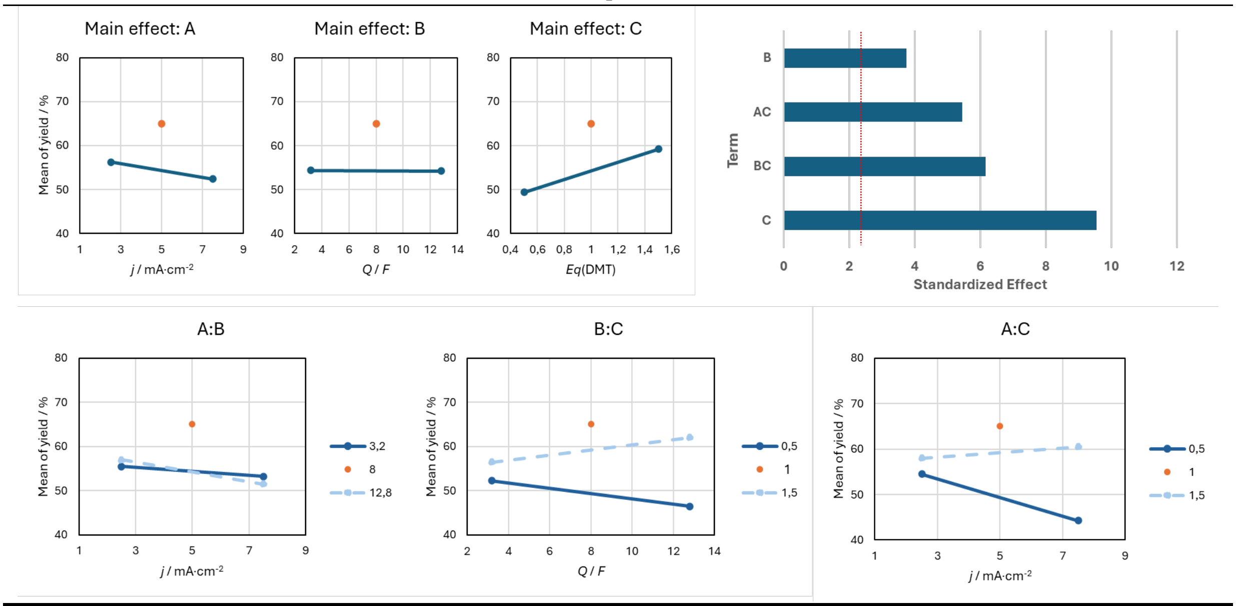

		Coded coefficients			
**Term**	**Effect**	**Coef**	**SE Coef**	***t*-value**	***p*-value**
const		0.543	0.0052	105.04	0.000
A	-0,03875	-0.019	0.0052	-3.75	0.005
B	-0,00125	-0.001	0.0052	-0.12	0.906
C	0,09875	0.049	0.0052	9.55	0.000
AB	-0,01625	-0.008	0.0052	-1.57	0.151
BC	0,05625	0.028	0.0052	5.44	0.000
AC	0,06375	0.032	0.0052	6.16	0.000
ABC	0,03125	0.016	0.0052	3.02	0.014
CtrPt		0.107	0.0155	6.89	0.000

After the evaluation of the 2^3 full factorial experiment design a surface response experimental design was added and a target value optimization for obtained GC yield of (**3**) was performed that predicted parameter values for further experiment optimization.

The target value optimization suggested the following adjustments of applied charge, current density and mediator concentration to increase the GC yield of (**3**) further. (j = 10 mA·cm^{-2}, Q = 17.2 *F*, c(DMT) = 0.1 M (1.25 Eq.)). Applying these conditions led to an increase in GC yield of (**3**) from an initial 63% to 74%. These new conditions with a hypothesized higher chlorine transfer rate were named DoE-Conditions.

Design of Experiments Conditions and Control Experiment

<u>DoE-conditions:</u> The second optimization by DoE-approach.

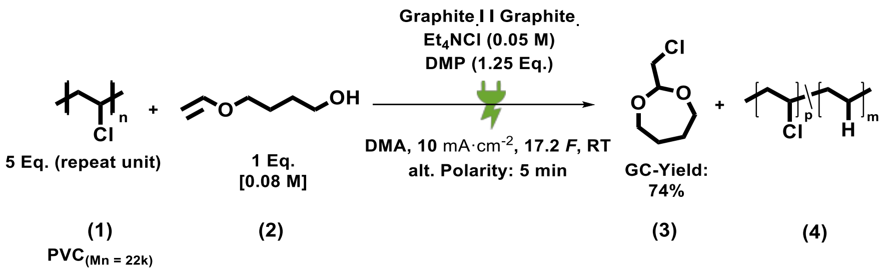

The GC yield of 2-chloromethyl-1,3-dioxepane (**3**) was increased from 63% to 74% using a stoichiometry of PVC **(1)** (repeat unit) to chlorine acceptor (**2**) of 5:1.

<u>Control experiment:</u> The electrolysis without PVC led to a GC yield of 9%. Confirming that only small amounts of chlorine atoms from the supporting electrolyte are used for product formation of (**3**).

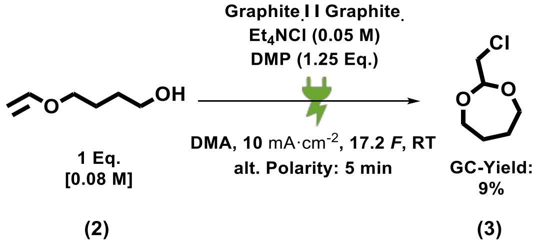

General procedures

General procedure A for the electrolysis in 5 mL IKA electrochemical cell (Optimized Conditions)

The polymer material PVC (100 mg, 1.6 mmol, 8 eq.(repeat unit)), the supporting electrolyte tetraethylammonium chloride (33.1 mg, 0.2 mmol, 1 eq, 0.05 M), dimethyl phthalate (38.8 mg, 0.2 mmol, 1 eq.) and 4-hydroxybutyl vinyl ether (23.2 mg, 25 µL, 0.2 mmol, 1 eq.) were transferred into the electrochemical cell (ElectraSyn® 5mL) and dissolved in *N*,*N*-dimethylacetamide (4 mL). The reaction mixture was stirred (500 rpm) for 15 minutes at room temperature using a Teflon-coated magnetic stir bar. The electrodes and the teflon cap were attached to the reaction vessel and the electrolysis was started. ($Q = 8\ F$, $j = 5\ mA{\cdot}cm^{-2}$, alt. Polarity = 15 min ($4\ h^{-1}$), electrode surface area ca. $3.0\ cm^{-2}$). After the reaction was finished, the internal standard 1,10-dichlorodecane (50 µL) was added and stirred for 5 minutes. For yield/conversion determination, samples for gas chromatography were prepared. For polymer analysis, the solution was added dropwise into methanol (50 mL) to induce polymer precipitation. After centrifugation the obtained polymer was redissolved in Tetrahydrofuran (3 mL) and precipitated again in methanol. After centrifugation the polymer was dried *in vacuum* and obtained as amorphous, colorless solid.

General procedure B for the electrolysis in 5 mL IKA electrochemical cell (DoE Conditions)

The polymer material PVC (100 mg, 1.6 mmol, 5 eq.(repeat unit)), the supporting electrolyte tetraethylammonium chloride (33.1 mg, 0.2 mmol, 0.625 Eq, 0.05 M), dimethyl phthalate (77.7 mg, 0.4 mmol, 1.25 eq.) and 4-hydroxybutyl vinyl ether (37.2 mg, 38 µL, 0.32 mmol, 1 eq.) were transferred into the electrochemical cell (ElectraSyn® 5mL) and dissolved in *N*,*N*-dimethylacetamide (4 mL). The reaction mixture was stirred (500 rpm) for 15 minutes at room temperature. The electrodes and the teflon cap were attached to the reaction vessel and the electrolysis was started. ($Q = 17.2\ F$, $j = 10\ mA{\cdot}cm^{-2}$, alt. Polarity = 5 min ($4\ h^{-1}$), electrode surface area ca. $3.0\ cm^{-2}$) After the reaction was finished, the internal standard 1,10-dichlorodecane (50 µL) was added and the solution was stirred for 5 minutes. For yield/conversion determination, samples for gas chromatography were prepared. For polymer analysis, the solution was added dropwise into methanol (50 mL) to induce polymer precipitation. After centrifugation the obtained polymer was redissolved in Tetrahydrofuran (3 mL) and precipitated again in methanol. After centrifugation the polymer was dried *in vacuum* and obtained as amorphous, colorless solid.

<u>Isolation of 2-Chloromethyl-1,3-dioxepane **(3):**</u> After the electrolysis was finished the reaction solution was added dropwise into methanol. The precipitated polymer was centrifuged, and the solution was transferred into a round bottom flask. Methanol was removed under reduced pressure. Lithium chloride solution (5%) was added to the residual mixture and the aqueous solution was extracted with diethyl ether. The organic phase was extracted with 5% lithium chloride solution and dried with $MgSO_4$. After filtration, the solvent was removed *in vacuo* and obtained the product via distillation at 82 °C/22 mbar. (Colorless liquid, isolated yield of 53%)

Polymer Analysis

After the development of the Optimized and DoE Conditions the obtained dechlorinated PVC was analyzed using size exclusion chromatography, NMR Spectroscopy, thermogravimetric analysis, differential scanning calorimetry and elemental analysis.

Size exclusion chromatography

The SEC elugram of the starting material shows a shoulder towards lower molecular weights. After the electrolysis using the optimized conditions the peak of the main species shifts to lower molecular weights, and the shoulder intensifies.

The defunctionalization leads to a poly(ethylene) subunit with different swelling behavior in DMF. The electrochemical backbone chain scission at random positions is less likely as no low molecular weight fragments or oligomers are visible in the elugram. Additionally, there are no species of higher molecular weights observed, hence recombination of polymer backbone radicals does not occur during electrolysis.

NMR Spectroscopy

Deuterated *d8*-Tetrahydrofuran was used to record the ^{1}H-NMR spectra of the starting material PVC. The polymer material became soluble in d_2-Tetrachlorethane after electrolysis, which was preferred for spectra evaluation due to less signal overlap.

It was shown that the phthalate esters are not electrochemically stable during the electrolysis with PVC. McNeill *et al.* showed that bulk electrolysis of di(2-ethylhexyl) phthalate (DEHP) showed the cleavage of the molecule's ester bonds leading to the detection of the alcohol (2-ethylhexanol). Due to the use of Dimethyl phthalate (DMP) in the electrolysis the generated methoxy species react with the polymer backbone leading to methoxy functionalization of the polymer.[2]

Multiple Electrolysis cycles

After the first electrolysis the polymer material was precipitated in MeOH as described in general procedure B, dried *in vacuo* and was redissolved in DMA for the next electrolysis reaction, applying again DoE conditions. This has been repeated four times. After each cycle the GC yield of 2-chloromethyl-1,3-dioxepane **(3)** has been determined.

Table S15: GC yields of 2-chloromethyl-1,3-dioxepane (3) of multiple electrolysis cycles using the PVC material as chlorine source four times. GC yields determined via gas chromatography using 1,10-dichlorodecane as internal standard.

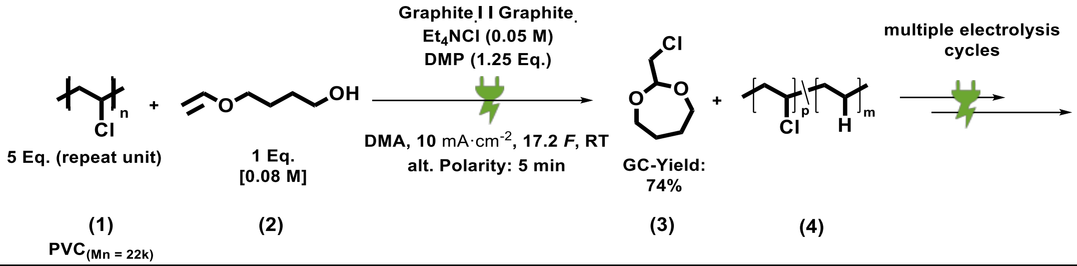

Multiple electrolysis cycles	GC yield (2-chloromethyl-1,3-dioxepane **(3)**) / %
1st cycle	74
2nd cycle	57
3rd cycle	32
4th cycle	17

GC yield, 1,10-dichlorodecane as internal standard.

The obtained polymer after the forth electrolysis cycle was soluble in MeOH and had to be precipitated in a mixture of cold hexane:diethyl ether (1:1).

In SEC chromatography no higher molecular weight species are observed that result out of uncontrolled radical backbone formation and network formation. ^{1}H NMR spectroscopy shows that the chemical nature of the starting material has been modified extensively, while the original PVC peak at a chemical shift of $\delta = 4.2–4.6$ ppm is not observable. This observation suggests a particularly high dechlorination rate of the polymer material using the method of multiple electrolysis cycles.

Thermogravimetric analysis (TGA)

To learn more about the dechlorination rates of the electrochemically treated PVC material TGA measurements were executed. In general, PVC is thermally instable, releasing HCl at around 280 °C. The second mass loss occurs around 450 °C indicates hydrocarbon backbone degradation. The theoretical mass loss of pure PVC due to HCl release is expected to be 58wt% leaving 42wt% of hydrocarbon material behind. The starting material PVC **(1)** (Sample 1, **a)**) reveals a mass loss of 61% at 280 °C. This mass loss is particularly sharp due to the zipper mechanism of thermal PVC elimination.[3] If we compare this sample with the electrochemically dechlorinated samples the mass loss at 280 °C decreases and broadens, likely due to polymer inhomogeneity and dechlorination.

The trend of decreased mass loss at 280 °C from optimized **(b)** via DoE conditions **(c)** to multiple electrolysis cycles **(d)** agrees with the findings from ^{1}H NMR spectroscopy and elemental analysis going down to a 11% mass loss for sample 4 (multiple electrolysis cycles).

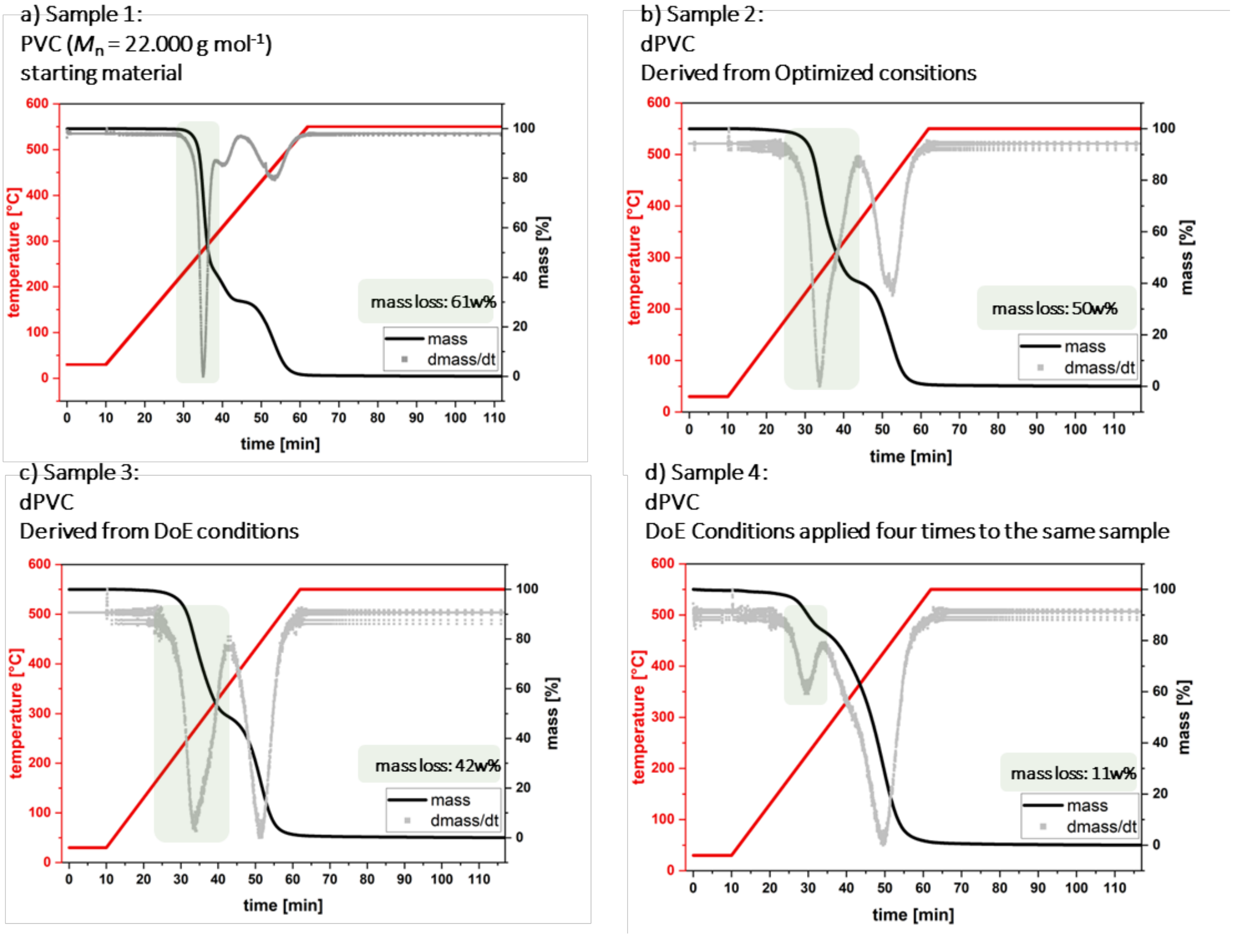

Figure S3: TGA data for the starting material (a) and the dechlorinated polymer samples. (b)- d))

NMR-spectroscopy showed significant amount of methoxy recombination of the polymer during the electrolysis. The elimination of the methoxy groups that could occur below 300 °C introduces distortions to the interpretation of the TGA data, which may result in erroneous conclusions regarding the degree of dechlorination. Consequently, supplementary elementary analyses were conducted.

Elemental analysis

Elemental analysis shows that the chlorine content decreases in all electrochemically treated samples. The optimized conditions lead to a dechlorination rate of 25% of PVC while the DoE conditions show more efficient dechlorination of 35%. The multiple treatment of PVC material applying the DoE conditions leads to a residual chlorine content of 3.58% after four electrolysis, yielding a PVC dechlorination rate of 94%.

Table S16: Elemental Analysis of the starting material, theoretical values and dechlorinated PVC samples.

Sample	%C	%H	%N	%O	%Cl	Sum / %
Starting Material – PVC (M_n = 22k)	37.96	4.88	0.00	0.82	55.98	99.64
PVC (theoretical)	38.46	4.83	0.00	0.00	56.71	100.00
Sample 2 - Optimized Conditions – dechlorinated PVC	44.84	5.27	0.43	6.38	42.35	99.27
DoE Conditions – dechlorinated PVC	48.19	5.39	0.15	9.28	36.36	99.37
DoE Conditions (Applied four times) – dechlorinated PVC	64.25	6.84	0.76	23.93	3.58	99.36

Elemental analysis of the starting material was executed and served as control experiment; the obtained result agrees well with the theoretical elemental composition.

Upscale and PVC (waste) products

Table S17: Screening of the current density for the optimization of the electrochemical reaction. GC yields of 2-chloromethyl-1,3-dioxepane (3) determined via gas chromatography using 1,10-dichlorodecane as internal standard.

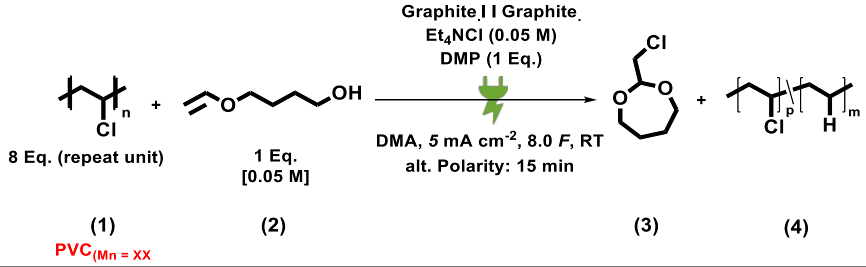

Polymer material	GC yield (2-chloromethyl-1,3-dioxepane **(3)**) / %	
	**Optimized Cond.**	**DoE-Conditions**
**5 mL vessel**, PVC (M_n = 22k)	77	74
**10 mL vessel**, PVC (M_n = 22k)	66	65
**250 mL vessel**, PVC (M_n = 22k)	-	34
5 mL vessel, PVC **(M_n = 99k)**	64	58
**1**-credit card	71	43
**2**-water hose	72	63
**3**-tube	68	56
**4**-cable channel	59	70
**5**-cable coating	65	51

GC yield, 1,10-dichlorodecane as internal standard.

Upscale experiments

For the upscale experiments general procedure B was executed while the weights and volumes of used chemicals were multiplied by the upscale factor (2x for the 10 mL vessel and 50x for the 250 mL vessel).

High molecular weight PVC

Since industrially produced PVC for everyday products has usually a higher molecular weight (approx. 100.000 g/mol), the influence of the molecular weight on the electrochemical reaction was investigated.

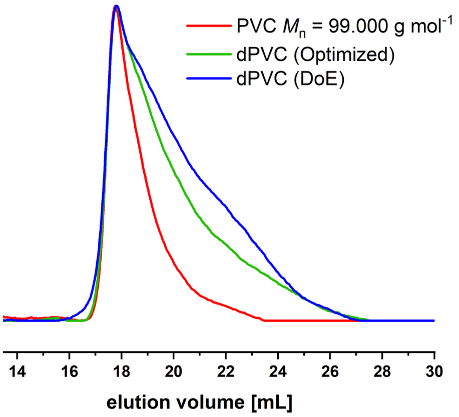

Figure S4: SEC elution traces of the starting material (*red*) and the dechlorinated PVC after electrolysis obtained from the optimized conditions (*green*), DoE conditions (*blue*). RI signal; eluent: DMF, 40 °C; standard: PMMA.

As discussed in section 2.4, the GC yield of the cyclic acetal decreases using high-molecular-weight PVC due to reasons such as lower average accessibility of carbon-chloride sites and increased viscosity of the reaction solution that decreases efficient mass transport during electrolysis. Therefore, we determined the viscosities of polymer solutions using PVC species with different molecular

weights. (Figure S5) In the left graph the viscosities of just the PVC polymer solutions in DMA were determined with the concentration how it's used in electrolysis (c = 0.4 M, PVC (repeat unit)). The polymer solution of low-molecular-weight PVC exhibits a viscosity of η = 4.5 Pa s, whereas the higher-molecular weight PVC solution gives an expected higher viscosity of η = 12.7 Pa s. In the right graph the viscosities of the electrolyte solutions (DoE conditions, c = 0.4 M, PVC (repeat unit)) additionally containing supporting electrolyte, the chlorine acceptor and the phthalate ester were determined. The obtained viscosities of the electrolyte solutions (PVC (22.000 g mol^{-1}) η = 4.4 Pa s, PVC (99.000 g mol^{-1}) η = 12.8 Pa s) are comparable with the viscosities obtained for solutions of PVC in DMA. These findings indicate that the molecular weight of the polymer PVC highly influences the viscosity of the reaction solution, while other components such as supporting electrolyte, phthalate ester and chlorine acceptor do not have a major impact on the solution viscosity.

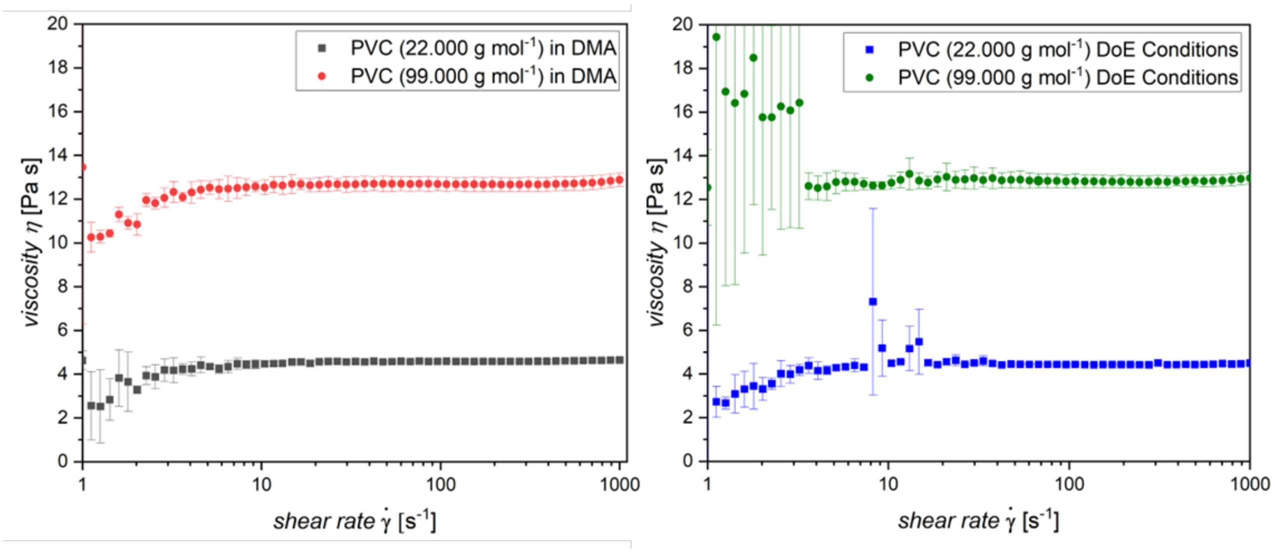

Figure S5: Determination of the viscosity of the polymer solutions containing different molecular weight PVC species. Left: PVC (c = 0.4 M, PVC (repeat unit)) in DMA using low-molecular-weight PVC (M_n = 22.000 g mol^{-1}, *black graph*) and high molecular weight PVC (M_n = 22.000 g mol^{-1}, *red graph*). Right: Solutions used for electrolysis (DoE conditions) with Et_4NCl (33.1 mg, 0.2 mmol, 0.625 Eq, 0.05 M), dimethyl phthalate (77.7 mg, 0.4 mmol, 1.25 eq.) and 4-hydroxybutyl vinyl ether (37.2 mg, 38 µL, 0.32 mmol, 1 eq.) using low-molecular-weight PVC (M_n = 22.000 g mol^{-1}, *blue graph*) and high molecular weight PVC (M_n = 22.000 g mol^{-1}, *black graph*).

Table S18: Overview of the viscosity measurements.

	η (PVC (M_n = 22.000 g mol^{-1})) / Pa s	η (PVC (M_n = 99.000 g mol^{-1})) / Pa s
In DMA (c = 0.4 M, PVC (repeat unit))	4.5	12.7
DoE Conditions (c = 0.4 M, PVC (repeat unit))	4.4	12.8

Analysis of PVC waste products

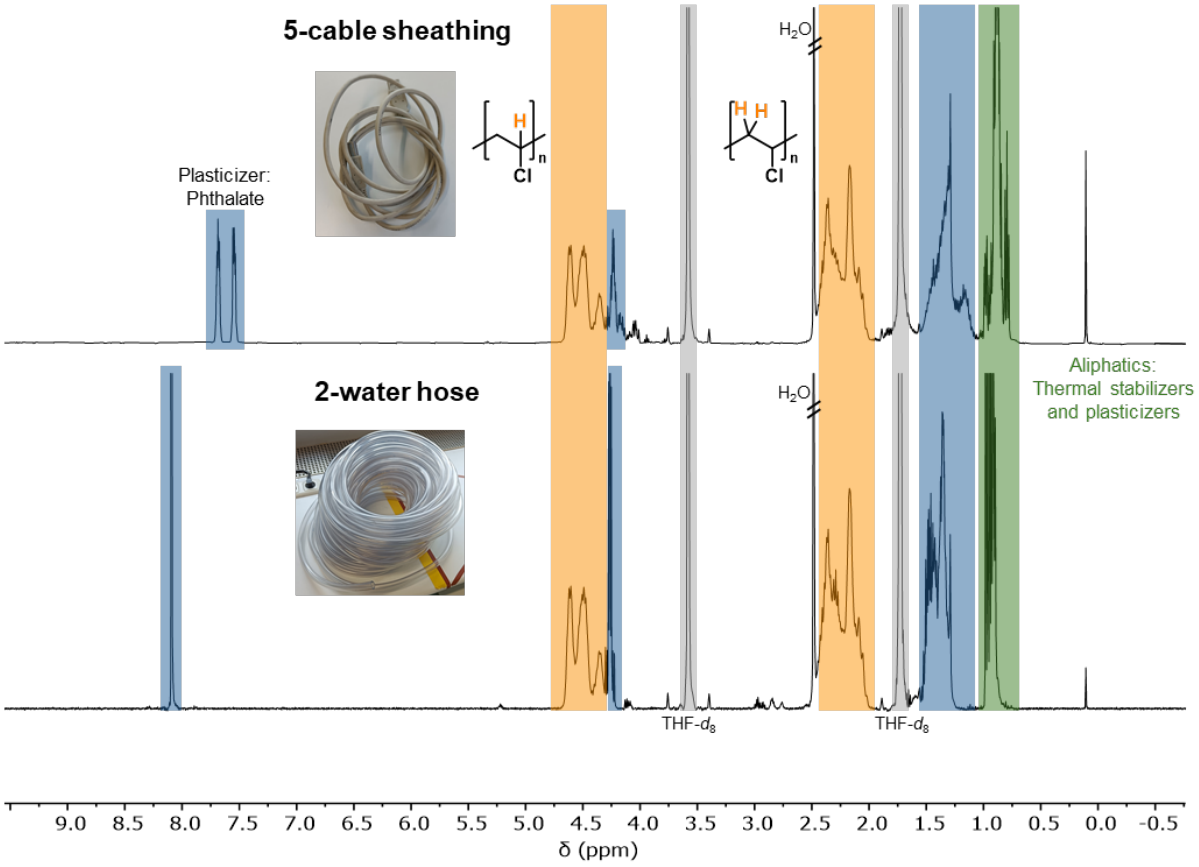

Figure S6: ^{1}H NMR of PVC products. (400 MHz, THF-d_8, 298 K)

Cyclic acetal scope

Overview

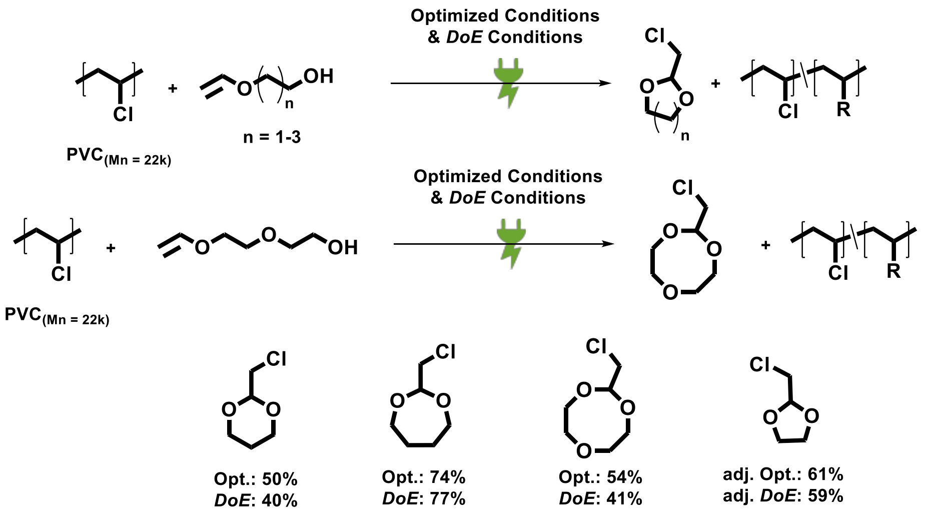

Synthesis of 2-chloromethyl-1,3-dioxolane (5-membered Ring)

The starting material ethyleneglycol monovinylether and the product 2-chloromethyl-1,3-dioxolane for GC-calibration were purchased commercially.

Ethyleneglycol monovinylether

^{1}H-NMR (400 MHz, C*D*$_2$Cl$_2$, 298 K): *δ*/ppm = 6.50 (dd, *J* = 14.3, 6.8 Hz, 1H, C**H**-O), 4.27 – 3.98 (m, 2H, C**H$_2$**), 3.78 (d, *J* = 4.0 Hz, 4H, O-**CH$_2$**), 2.16 – 2.08 (m, 1H, O**H**).

^{13}C-NMR (101 MHz, C*D*Cl$_3$, 298 K): *δ*/ppm = 152.06 (H**C**=CH$_2$), 87.10 (HC=**C**H$_2$), 69.83 (O-**C**H$_2$), 61.51 (**C**H$_2$-OH).

2-Chloromethyl-1,3-dioxolane

^{1}H-NMR (400 MHz, C*D*Cl$_3$, 298 K): *δ*/ppm = 5.16 (t, J = 4.0 Hz, 1H, O-C**H**-O), 4.07 – 3.92 (m, 4H, O-C**H$_2$**), 3.54 (d, J = 4.0 Hz, 2H, C**H$_2$**-Cl).

^{13}C-NMR (101 MHz, C*D*Cl$_3$, 298 K): *δ*/ppm = 102.78 (O-**C**H-O), 65.96 (O-**C**H$_2$), 45.04 (**C**H$_2$-Cl).

GC-MS (Std. method, column: Rxi-5Sil MS): t_R = 3.70 min.

GC (Std. method, column: ZB-5): t_R = 4.84 min.

GC calibration for yield determination of 2-chloromethyl-1,3-dioxolane

Table S19: GC calibration for yield determination of 2-chloromethyl-1,3-dioxolane (5) using 1,10-dichlorodecane (1,10-DD) as internal standard.

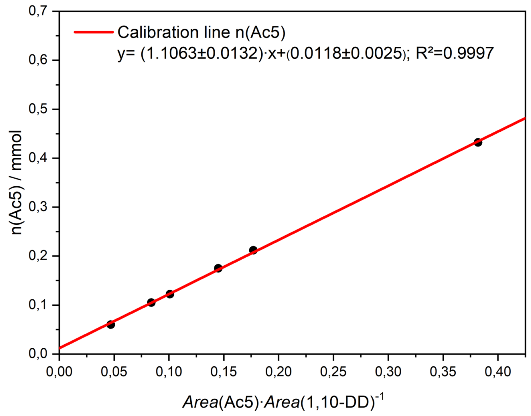

A(Ac5)·A^{-1}(1,10-dichlorodecane)	n(Ac5) / mmol
0	0.016
0.015	0.053
0.017	0.060
0.030	0.083
0.051	0.108
0.089	0.142
0.092	0.168
0.225	0.309

GC yield, 1,10-dichlorodecane as internal standard.

The general procedures A & B using the starting material instead of **(2)** were applied to test the transferability towards different ring sizes. Due to the very low GC-yields the optimized and DoE conditions were adjusted: The supporting electrolyte Oct_3MeNCl (0.01 M) was used with a smaller applied charge. These changes led yields of 61% and 59% for the optimized conditions and the DoE conditions.

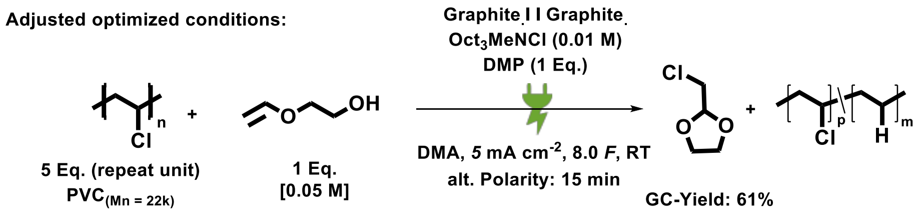

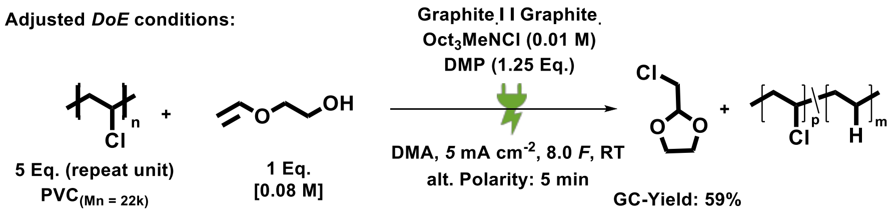

Synthesis of 2-chloromethyl-1,3-dioxane (6-membered ring)

The starting material 1-vinyl-propandi-1,3-ol[4] and the product 2-chloromethyl-1,3-dioxane[1] for the electrochemical cyclic acetal formation have been synthesized according to the following procedures:

Classical synthesis of 1-vinyl-propandi-1,3-ol

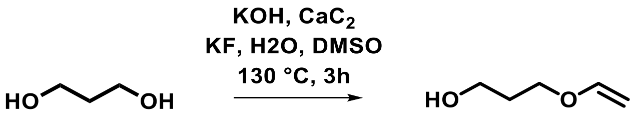

A pressure pipe flask (25 mL) was charged with potassium hydroxide (29 mmol, 1.63 g, 1 eq.), 1,3-propanediol (29 mmol, 2.21 g, 2.1 mL, 1 eq.), potassium fluoride (116 mmol, 6.70 g), and calcium carbide (58 mmol, 3.70 g). Dry DMSO (10 mL) was then added, and the flask was sealed with a septum. After stirring at room temperature for 5 minutes, water (120 mmol, 2.2 mL) was introduced through the valve. The mixture was subsequently heated to 130 °C with vigorous stirring for 3 h. Upon cooling to 25 °C, the reaction mixture was extracted with diethyl ether (3 × 50 mL). The combined organic layers were concentrated under reduced pressure to give 1-vinyl propanedi-1,3-ol as a colorless oil. (21%)

^{1}H-NMR (400 MHz, C*D*Cl$_3$, 298 K): *δ*/ppm = 6.43 (dd, *J* = 14.3, 6.8 Hz, 1H, C**H**=CH$_2$), 4.18 (dd, *J* = 14.3, 2.1 Hz, 1H, CH=C**H$_2$**), 3.98 (dd, *J* = 6.8, 2.1 Hz, 1H, CH=C**H$_2$**), 3.80 (t, *J* = 6.0 Hz, 2H, =CH$_2$-O-C**H$_2$**), 3.73 (q, *J* = 5.7 Hz, 2H, C**H$_2$**-OH), 2.42 (t, *J* = 5.2 Hz, 1H, CH$_2$-O**H**), 1.88 (p, *J* = 6.0 Hz, 2H, CH$_2$-C**H$_2$**-CH$_2$).

^{13}C-NMR (101 MHz, C*D*Cl$_3$, 298 K): δ/ppm =

GC-MS (Std. method, column: Rxi-5Sil MS): t_R = 3.13 min.

GC (Std. method, column: ZB-5): t_R = 4.32 min.

Classical synthesis of the 2-chloromethyl-1,3-dioxane

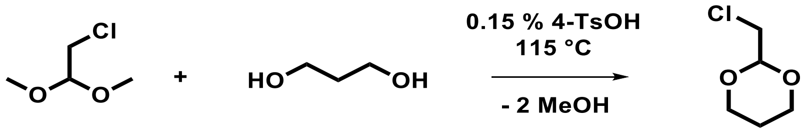

2-Chloromethyl-1,3-dioxane was synthesized by fractional vacuum distillation. 1,3-propanediol (10.02 g, 0.13 mol, 1 eq.), chloroacetaldehyde dimethylacetal (16.41 g, 0.13 mol, 1 eq.) and 4-toluenesulfonic acid (38 mg, 0.203 mmol, 0.002 eq.) were added to in a round bottom flask. The reaction mixture was heated under normal pressure and MeOH was distilled off at 58 °C. Fractional distillation was then carried out at 58 mbar. The colorless product was obtained in the collecting flask at a temperature of 30 °C. (77%)

^{1}H-NMR (400 MHz, C*D*Cl$_3$, 298 K): *δ*/ppm = 4.69 (t, *J* = 4.5 Hz, 1H, O-C**H**-O), 4.11 (ddd, *J* = 12.0, 5.0, 1.4 Hz, 2H, O-C**H$_2$**), 3.85 – 3.73 (m, 2H, O-C**H$_2$**), 3.45 (d, *J* = 4.5 Hz, 2H, Cl-C**H$_2$**), 2.05 (dtt, *J* = 13.5, 12.5, 5.0 Hz, 1H, C**H**$_2$), 1.35 (dtt, *J* = 13.5, 2.6, 1.4 Hz, 1H, C**H**$_2$).

^{13}C-NMR (101 MHz, C*D*Cl$_3$, 298 K): δ/ppm = 100.03 (O-**C**H-O), 66.95 (O-**C**H$_2$), 44.69 (Cl-**C**H$_2$), 25.50 (**C**H$_2$).

GC-MS (Std. method, column: Rxi-5Sil MS): t_R = 4.70 min.

GC (Std. method, column: ZB-5): t_R = 6.44 min.

GC calibration for yield determination of 2-chloromethyl-1,3-dioxane

Table S20: GC calibration for yield determination of 2-chloromethyl-1,3-dioxane (6) using 1,10-dichlorodecane (1,10-DD) as internal standard.

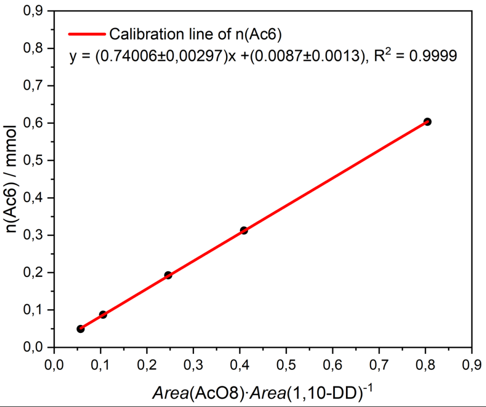

$A(Ac6)\cdot A^{-1}$(1,10-dichlorodecane)	n(Ac6) / mmol
0.106	0.087
0.057	0.049
0.246	0.192
0.409	0.313
0.805	0.603

GC yield, 1,10-dichlorodecane as internal standard.

Synthesis of 2-chloromethyl-[1,3,6] trioxocane (8-membered ring)

The starting material di ethylene glycol mono vinyl ether was purchased commercially. The product was synthesized according to a synthesis protocol published from TARDY *et al.* The product was used for GC calibration and GC-MS identification.[1]

<u>Classical synthesis of of 2-chloromethyl-[1,3,6] trioxocane</u>

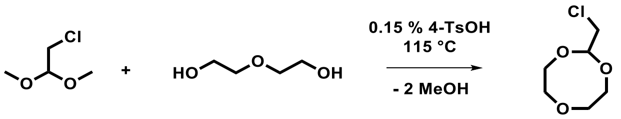

2-chloromethyl-1,3,6-trioxocane was synthesized by fractional vacuum distillation. Diethylene glycol (10.02 g, 0.094 mol, 8.95 mL, 1 eq.), chloroacetaldehyde dimethylacetal (11.74 g, 0.094 mol, 10.73 mL, 1 eq.) and 4-toluenesulfonic acid (28 mg, 0.147 mmol, 0.002 eq.) were added to in a round bottom flask. The reaction mixture was heated under normal pressure and MeOH was distilled off at 58 °C. Fractional distillation was then carried out at 57 mbar. The colorless product crystallized out in the condenser and in the collecting flask at a temperature of 30 °C.

^{1}H-NMR (400 MHz, C*D*Cl$_3$, 298 K): δ/ppm = = 4.78 (t, J = 5.4 Hz, 1H, O-C**H**-O), 4.05 – 3.91 (m, 4H, CH-O-C**H**$_2$), 3.83 – 3.61 (m, 4H, C**H**$_2$-O), 3.47 (d, J = 5.4 Hz, 2H, C**H**$_2$-Cl).

^{13}C-NMR (101 MHz, C*D*Cl$_3$, 298 K): δ/ppm = 106.47 (O-**C**H-O), 73.97 (CH-O-C**H**$_2$), 71.96 (O-**C**H$_2$), 45.49 (C**H**$_2$-Cl).

GC-MS (Std. method, column: Rxi-5Sil MS): t_R = 6.14 min.

GC (Std. method, column: ZB-5): t_R = 8.75 min.

GC calibration for yield determination of 2-chloromethyl-[1,3,6] trioxocane

Table S21: GC calibration for yield determination of 2-chloromethyl-[1,3,6] trioxocane (7) using 1,10-dichlorodecane (1,10-DD) as internal standard.

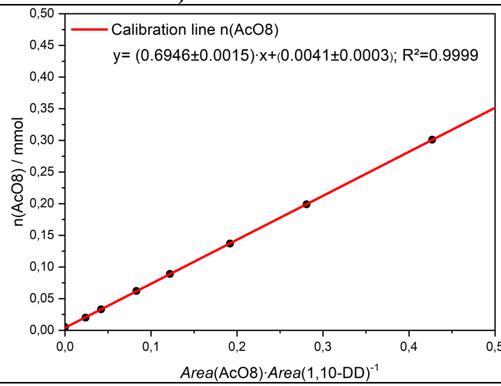

$A((7))\cdot A^{-1}$(1,10-dichlorodecane)	n((7)) / mmol
0	0.005
0.024	0.020
0.042	0.033
0.083	0.062
0.122	0.089
0.192	0.137
0.281	0.199
0.427	0.301

GC yield, 1,10-dichlorodecane as internal standard.

References

[1] A. Tardy, N. Gil, C. M. Plummer, D. Siri, D. Gigmes, C. Lefay, Y. Guillaneuf, *Angew. Chem. Int. Ed.* **2020**, *59*, 14517.

[2] D. E. Fagnani, D. Kim, S. I. Camarero, J. F. Alfaro, A. J. McNeil, *Nat. Chem.* **2023**, *15*, 222.

[3] J. Yu, L. Sun, C. Ma, Y. Qiao, H. Yao, *Waste Manag.* **2016**, *48*, 300.

[4] G. Werner, K. S. Rodygin, A. A. Kostin, E. G. Gordeev, A. S. Kashin, V. P. Ananikov, *Green Chem.* **2017**, *19*, 3032.

Spectra

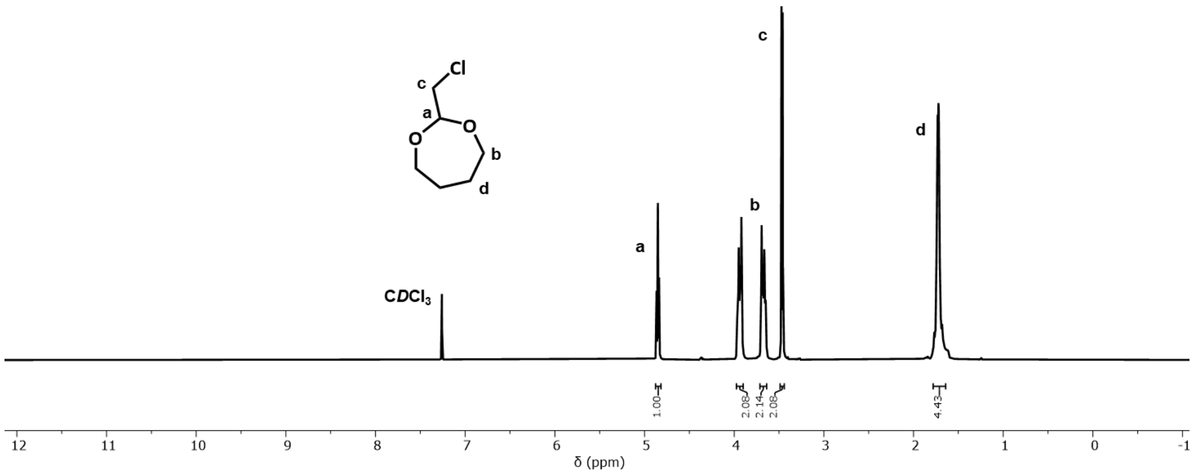

Figure S7: ^{1}H NMR spectrum of compound 3. (400 MHz, C*D*Cl_3, 298 K)

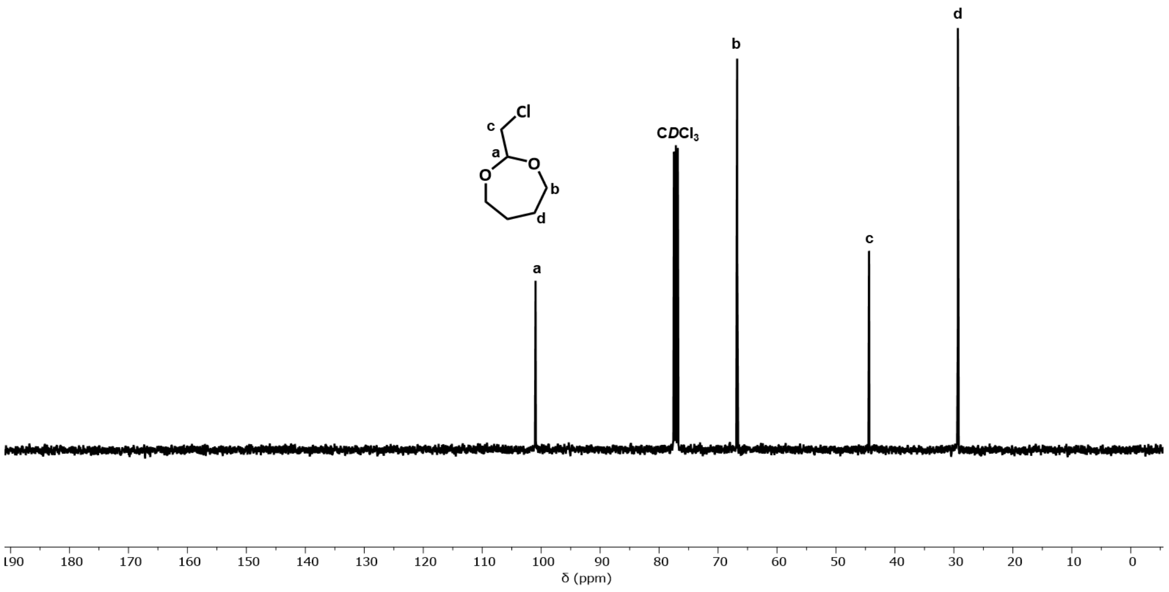

Figure S8: ^{13}C NMR spectrum of compound 3. (101 MHz, C*D*Cl_3, 298 K)

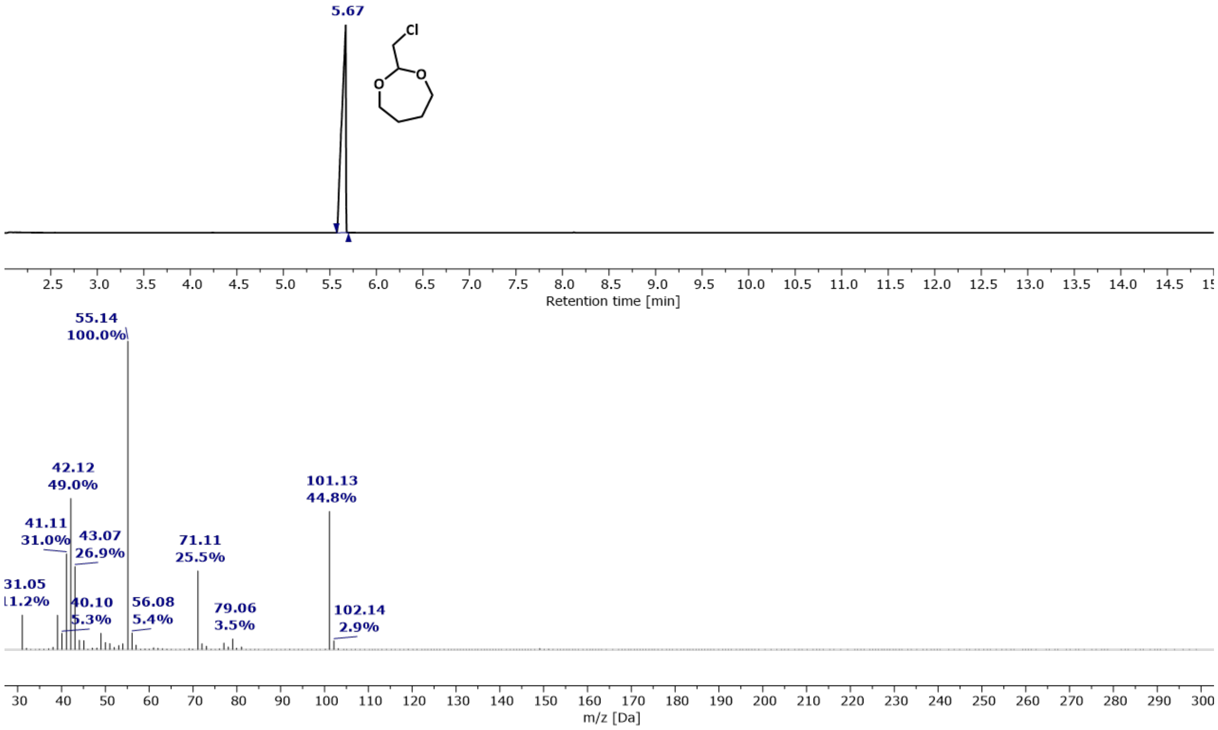

Figure S9: GC-MS trace and mass fragmentation pattern of compound 3.

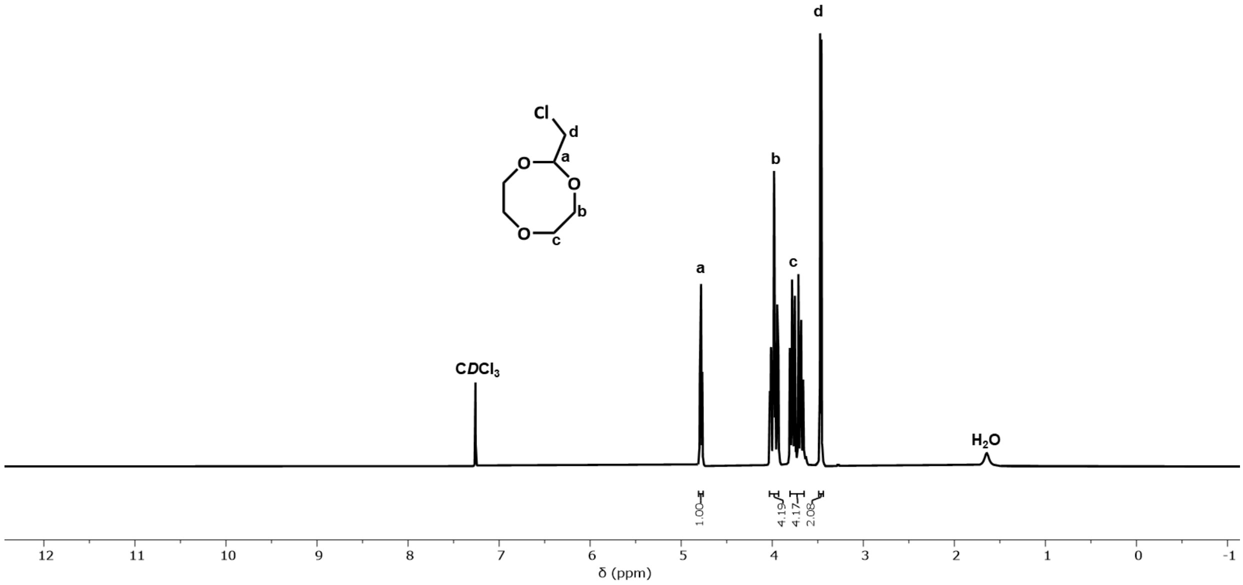

Figure S10: ^{1}H NMR spectrum of compound 7. (400 MHz, C*D*Cl$_3$, 298 K)

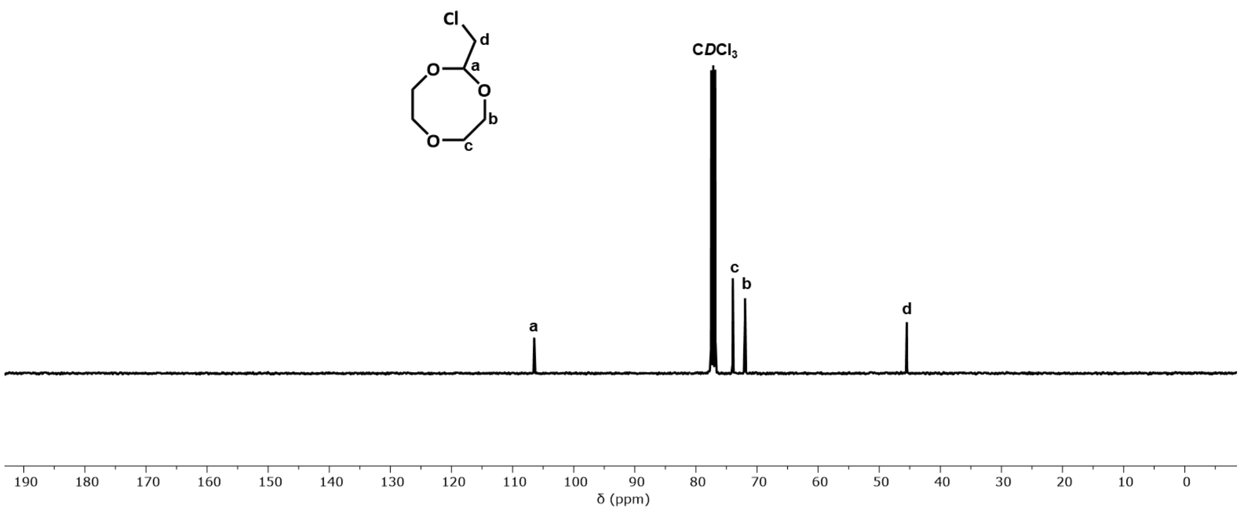

Figure S11: ^{13}C NMR spectrum of compound 7. (101 MHz, C*D*Cl$_3$, 298 K)

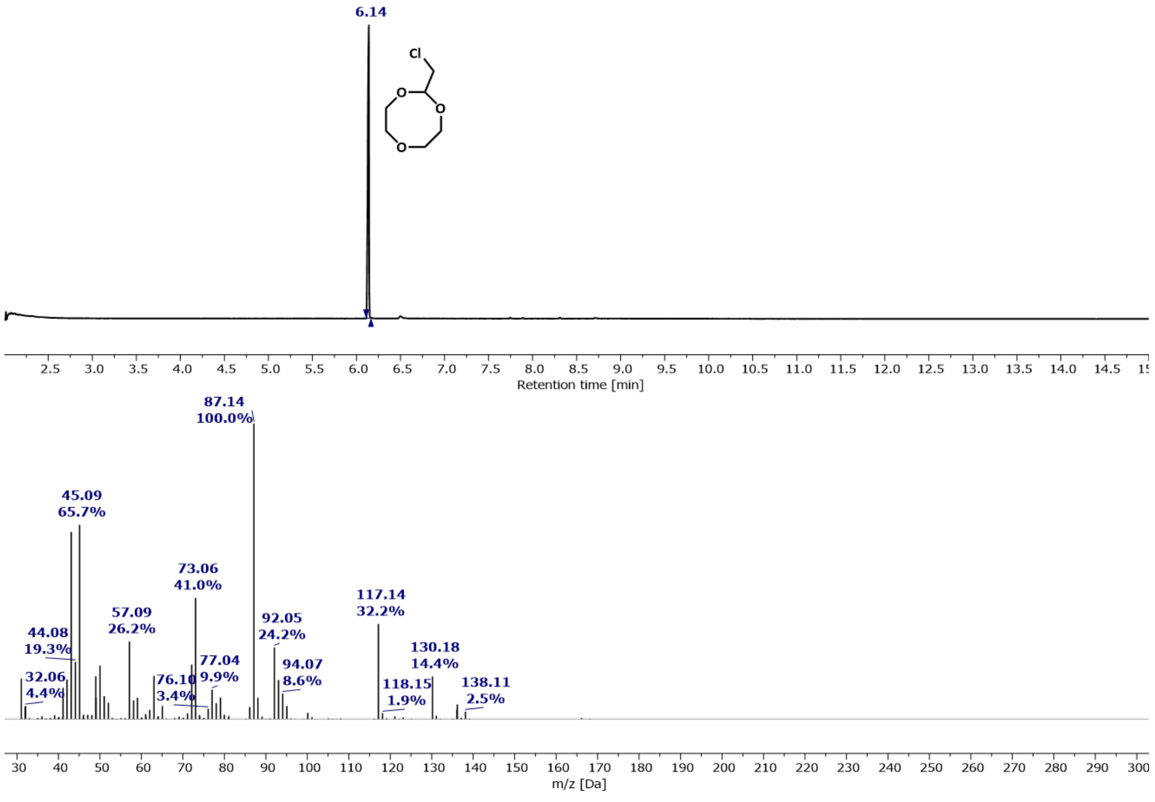

Figure S12: GC-MS trace and mass fragmentation pattern of compound 7.

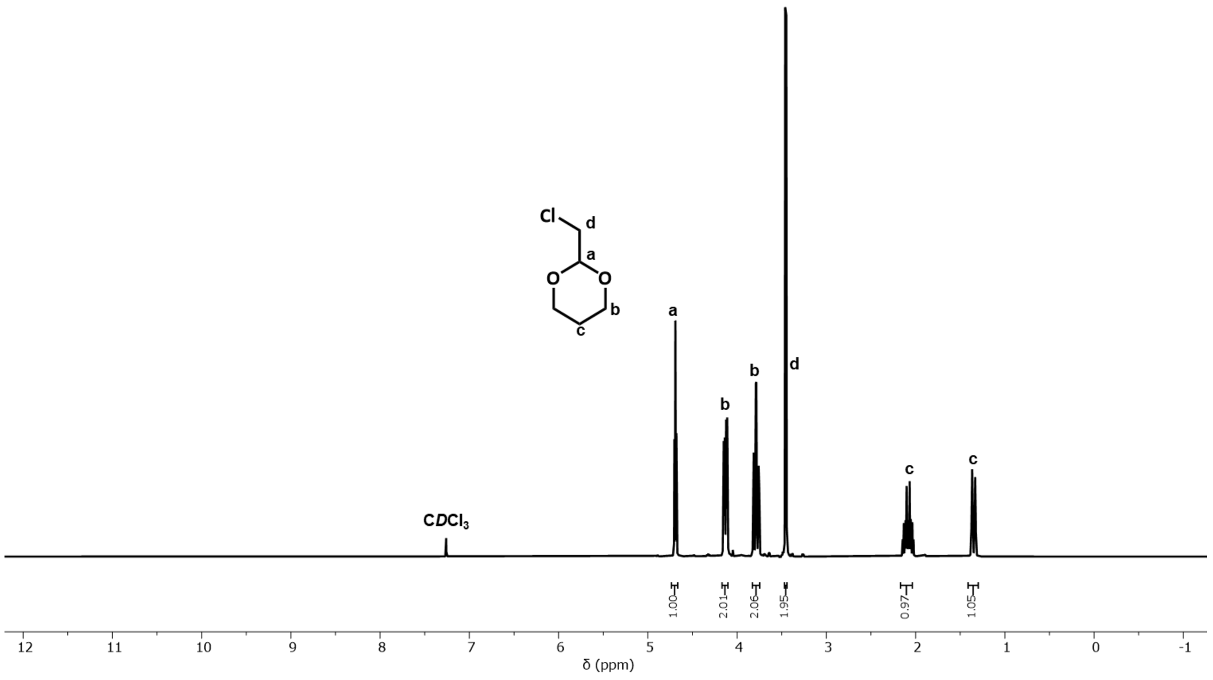

Figure S13: ^{1}H NMR spectrum of compound 6. (400 MHz, C*D*Cl$_3$, 298 K)

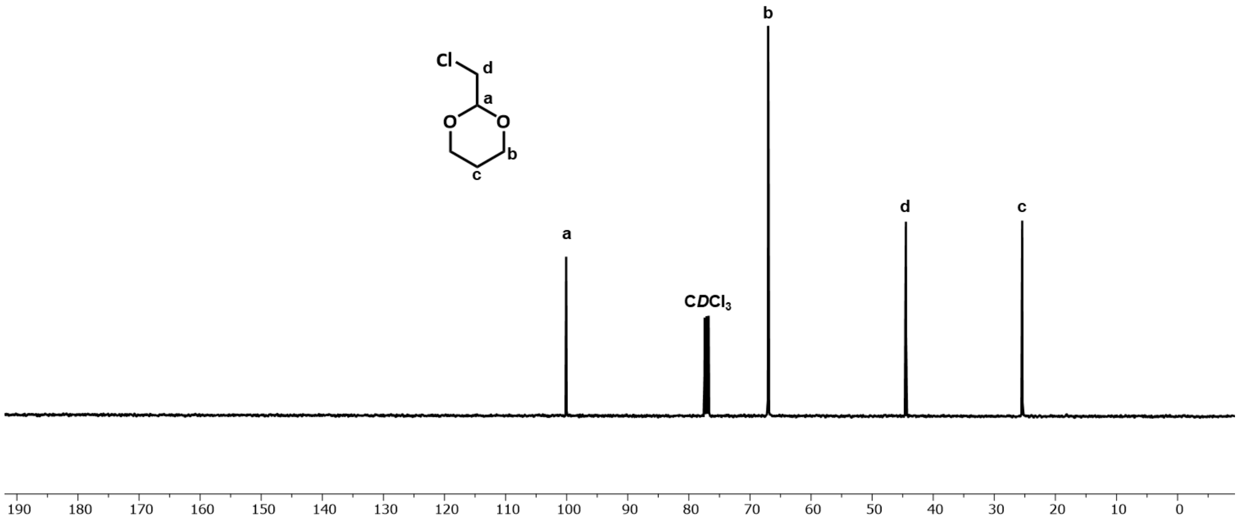

Figure S14: ^{13}C NMR spectrum of compound 7. (101 MHz, C*D*Cl$_3$, 298 K)

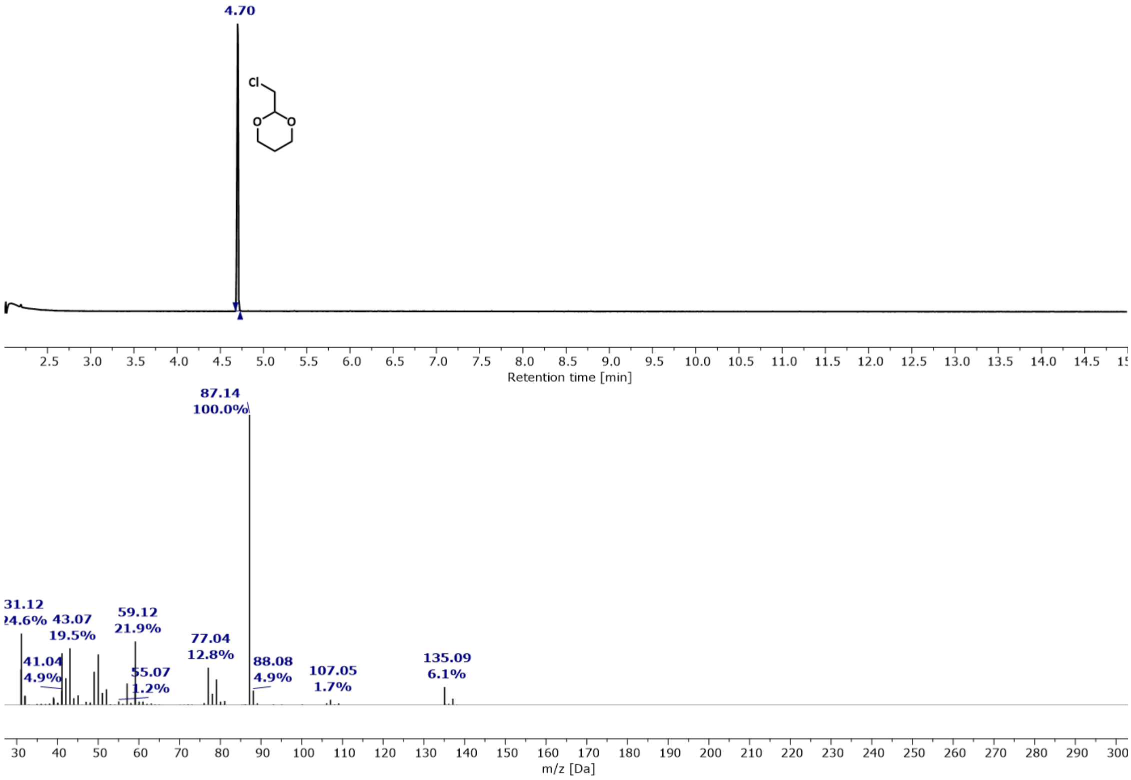

Figure S15: GC-MS trace and mass fragmentation pattern of compound 7.

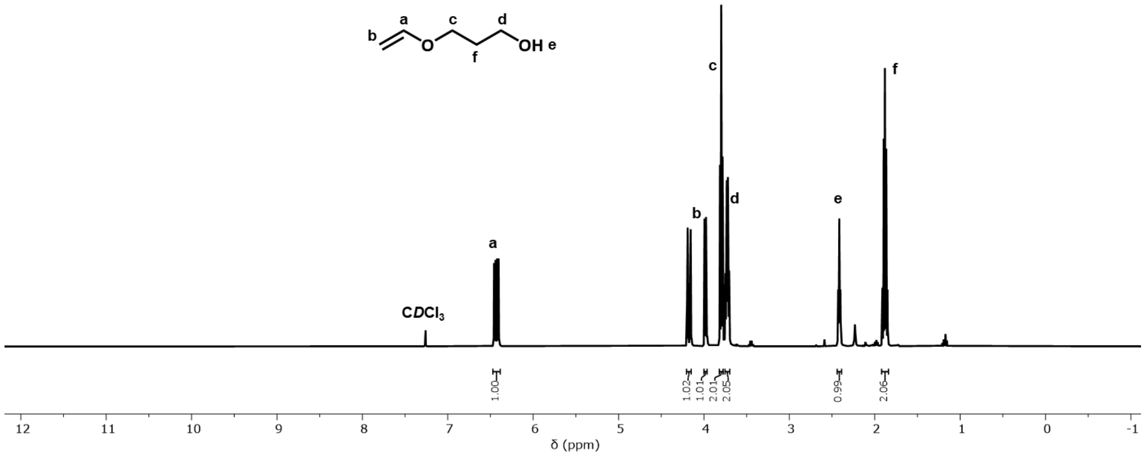

Figure S16: ^{1}H NMR spectrum of 3-hydroxypropyl vinyl ether. (400 MHz, C*D*Cl$_3$, 298 K)

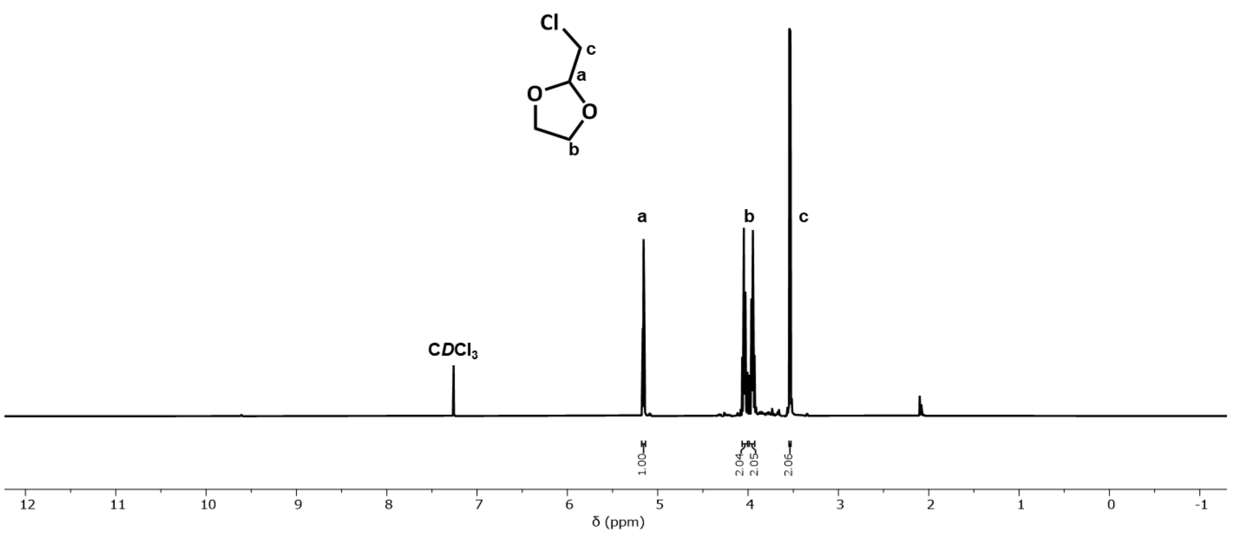

Figure S17: ^{1}H NMR spectrum of compound 5. (400 MHz, C*D*Cl$_3$, 298 K)

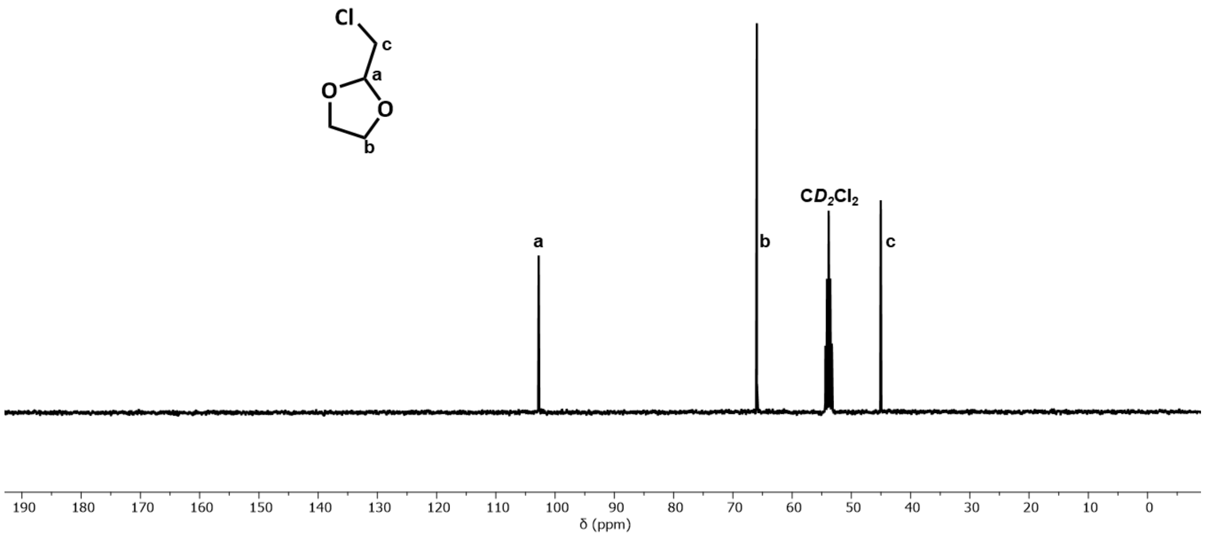

Figure S18: ^{13}C NMR spectrum of compound 5. (101 MHz, C*D*$_2$Cl$_2$, 298 K)

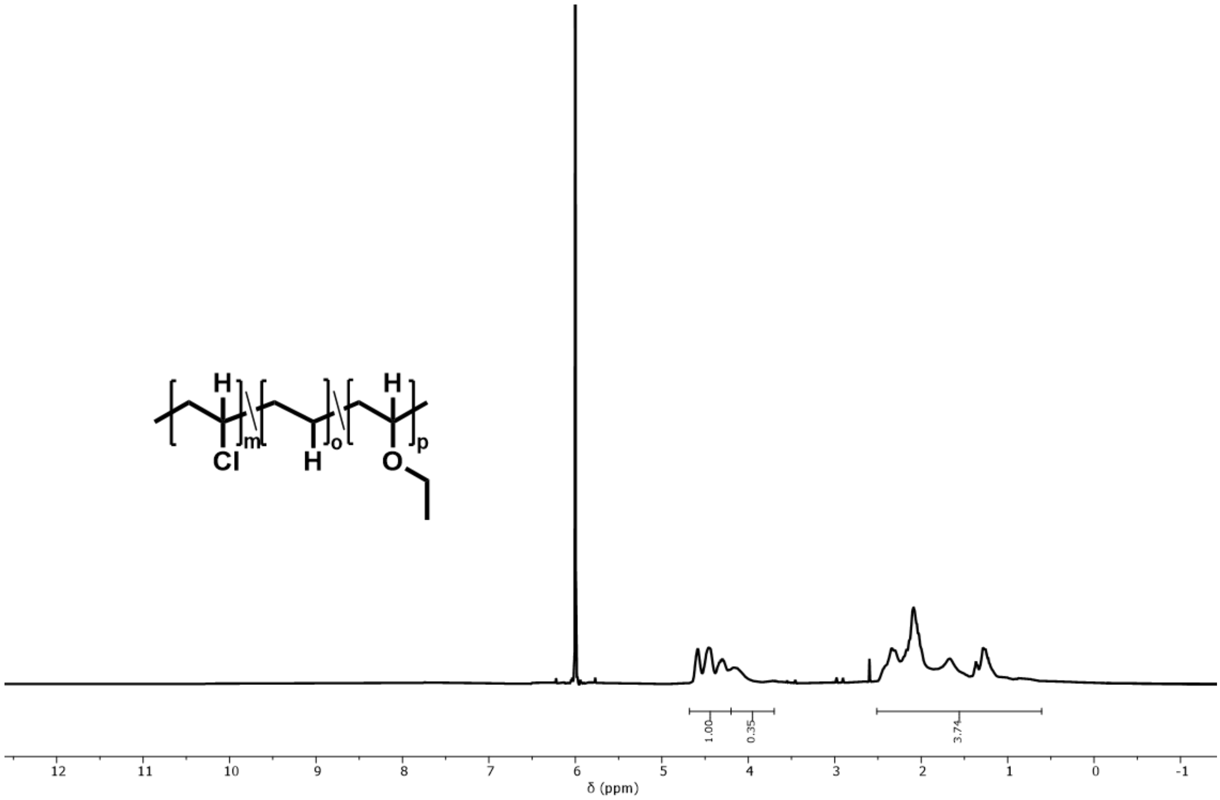

Figure S19: ^{1}H NMR spectrum of dPVC using diethyl phthalate in electrolysis applying DoE conditions. (400 MHz, TCE-d_2, 298 K)

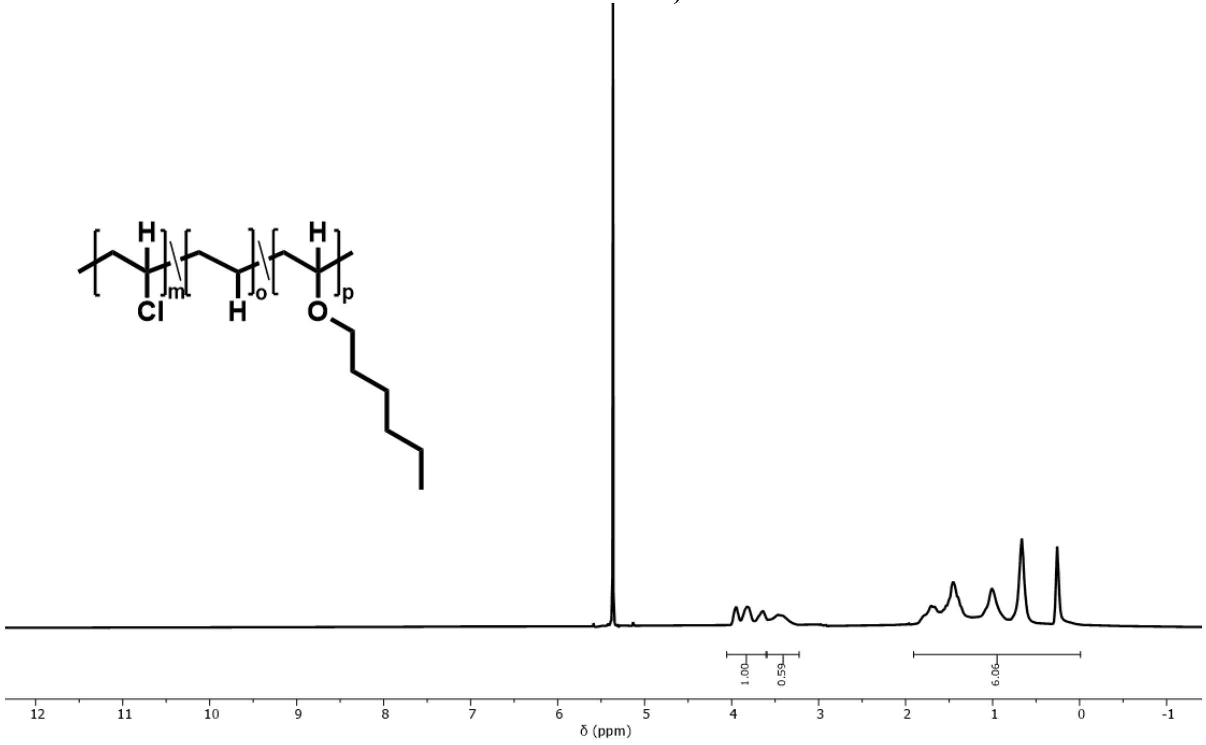

Figure S20: ^{1}H NMR spectrum of dPVC using dihexyl phthalate in electrolysis applying DoE conditions. (400 MHz, TCE-d_2, 298 K)

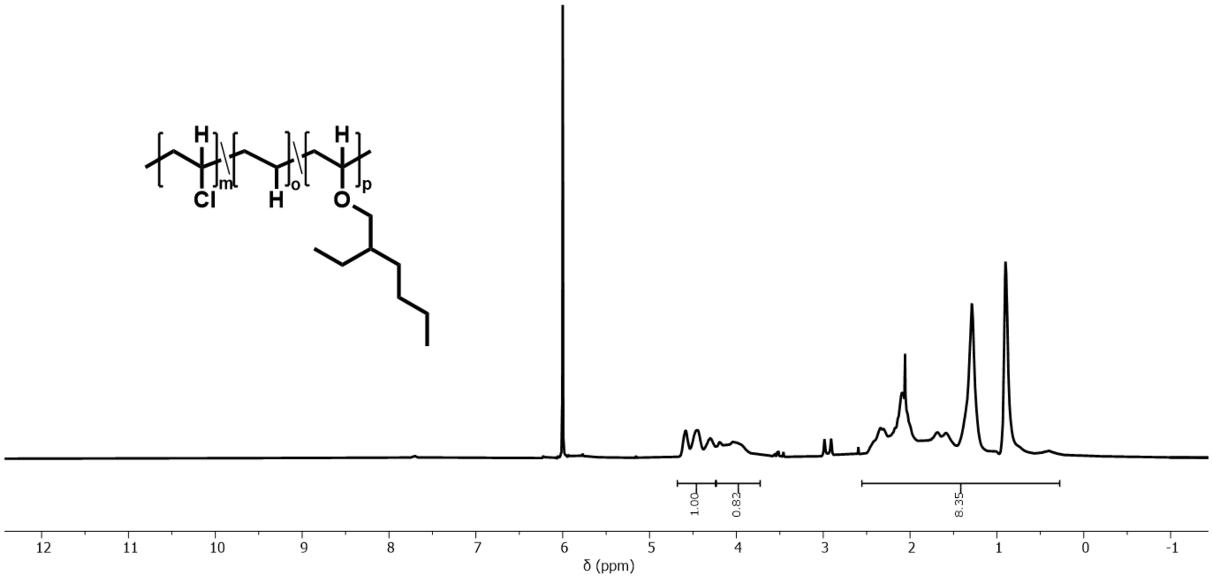

Figure S21: ^{1}H NMR spectrum of dPVC using bis(2-ethylhexyl) phthalate in electrolysis applying DoE conditions. (400 MHz, TCE-d_2, 298 K)

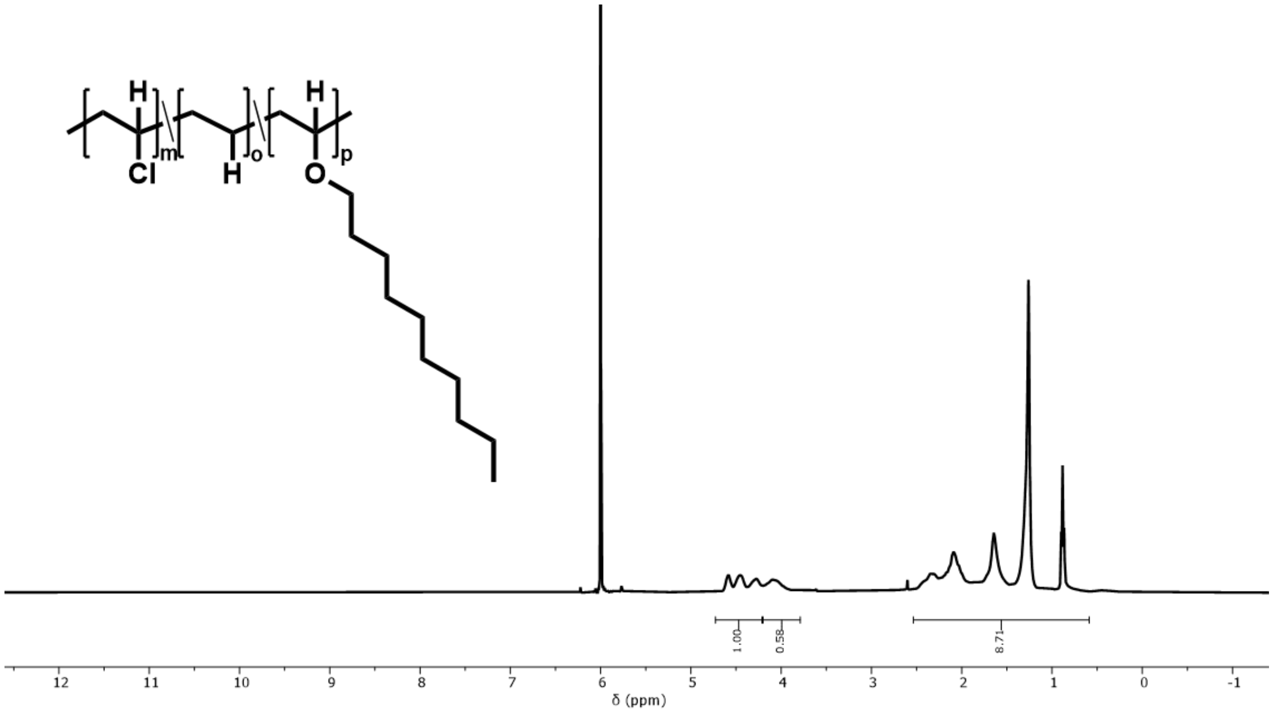

Figure S22: ^{1}H NMR spectrum of dPVC using didecyl phthalate in electrolysis applying DoE conditions. (400 MHz, TCE-d_2, 298 K)
